# Structure-guided antibody cocktail for prevention and treatment of COVID-19

**DOI:** 10.1371/journal.ppat.1009704

**Published:** 2021-10-21

**Authors:** Shih-Chieh Su, Tzu-Jing Yang, Pei-Yu Yu, Kang-Hao Liang, Wan-Yu Chen, Chun-Wei Yang, Hsiu-Ting Lin, Mei-Jung Wang, Ruei-Min Lu, Hsien-Cheng Tso, Meng-Jhe Chung, Tzung-Yang Hsieh, Yu-Ling Chang, Shin-Chang Lin, Fang-Yu Hsu, Feng-Yi Ke, Yi-Hsuan Wu, Yu-Chyi Hwang, I-Ju Liu, Jian-Jong Liang, Chun-Che Liao, Hui-Ying Ko, Cheng-Pu Sun, Ping-Yi Wu, Jia-Tsrong Jan, Yuan-Chih Chang, Yi-Ling Lin, Mi-Hua Tao, Shang-Te Danny Hsu, Han-Chung Wu

**Affiliations:** 1 Institute of Cellular and Organismic Biology, Academia Sinica, Taipei, Taiwan; 2 Institute of Biologic Chemistry, Academia Sinica, Taipei, Taiwan; 3 Institute of Biochemical Sciences, National Taiwan University, Taipei, Taiwan; 4 Biomedical Translation Research Center (BioTReC), Academia Sinica, Taipei, Taiwan; 5 Institute of Biomedical Sciences, Academia Sinica, Taipei, Taiwan; 6 Genomics Research Center, Academia Sinica, Taipei, Taiwan; The Peter Doherty Institute and Melbourne University, AUSTRALIA

## Abstract

Development of effective therapeutics for mitigating the COVID-19 pandemic is a pressing global need. Neutralizing antibodies are known to be effective antivirals, as they can be rapidly deployed to prevent disease progression and can accelerate patient recovery without the need for fully developed host immunity. Here, we report the generation and characterization of a series of chimeric antibodies against the receptor-binding domain (RBD) of the severe acute respiratory syndrome coronavirus 2 (SARS-CoV-2) spike protein. Some of these antibodies exhibit exceptionally potent neutralization activities *in vitro* and *in vivo*, and the most potent of our antibodies target three distinct non-overlapping epitopes within the RBD. Cryo-electron microscopy analyses of two highly potent antibodies in complex with the SARS-CoV-2 spike protein suggested they may be particularly useful when combined in a cocktail therapy. The efficacy of this antibody cocktail was confirmed in SARS-CoV-2-infected mouse and hamster models as prophylactic and post-infection treatments. With the emergence of more contagious variants of SARS-CoV-2, cocktail antibody therapies hold great promise to control disease and prevent drug resistance.

## Author summary

Effective approaches to mitigate the COVID-19 pandemic are a pressing global need. One promising strategy is to combine neutralizing antibodies that can reduce viral load to prevent disease progression and accelerate patient recovery. However, the current supply of therapeutic antibodies for COVID-19 is insufficient to fill the enormous demand, and escape mutants may compromise the utility of existing drugs. Thus, there is an urgent worldwide need to develop highly potent neutralizing antibody cocktails. We generated a series of chimeric antibodies against the receptor-binding domain (RBD) of SARS-CoV-2 spike protein, which potently neutralize authentic SARS-CoV-2 infection according to the plaque reduction neutralization test (PRNT) and pseudovirus-based inhibition assay. These antibodies can be classified into three distinct groups based on their targets within the receptor-binding motif. Cryo-electron microscopy structural analyses of two representative receptor-binding domain-chimeric antibodies in complex with the SARS-CoV-2 spike protein further revealed two sets of non-overlapping epitopes, suggesting the potential for their combination in a therapeutic antibody cocktail. The prophylactic and therapeutic effects of these antibodies and their combination were demonstrated in SARS-CoV-2-infected mouse and hamster models. Thus, our potent neutralizing antibody cocktail has strong potential for development as an effective therapeutic drug to prevent and treat SARS-CoV-2 infection.

## Introduction

Rapid spread of severe acute respiratory syndrome coronavirus 2 (SARS-CoV-2) has caused the coronavirus disease 2019 (COVID-19) pandemic, with more than 235 million people infected and 4.81 million dead as of by October 2021. In response to this dire situation, the global research community has made major efforts to develop novel treatments for prevention and therapy of COVID-19. These include vaccines that induce host immunity [1–5], small molecules that counteract pathological progression [6–11] and neutralizing antibodies (nAbs) that prevent the virus from entering host cells [12–18]. Experimental treatments based on COVID-19 convalescent sera, which contain nAbs, showed promising clinical effects that can help prevent progression to intensive care and mortality for infected individuals [19,20].

SARS-CoV-2 infection is initiated by the engagement of the spike (S) protein receptor-binding domain (RBD) to the host receptor molecule, angiotensin-converting enzyme 2 (ACE2). This binding triggers subsequent conformational changes within the S protein that enable viral entry. Most neutralizing antibodies (nAbs) therefore target the RBD to compete with ACE2 and prevent viral entry. Prior to the COVID-19 outbreak, several nAbs were developed for SARS-CoV and Middle East Respiratory Syndrome (MERS) CoV [21]. As SARS-CoV-2 and SARS-CoV share 74% sequence similarity in their RBDs, nAbs against SARS-CoV are potentially available for neutralizing SARS-CoV-2 [15,17]. Furthermore, single chain nanobodies from llamas that recognize SARS-CoV S RBD can also bind to the RBD of SARS-CoV-2 S protein with high affinities [22]. During the COVID-19 pandemic, major efforts have also been devoted to identifying nAbs from COVID-19 convalescent sera [12–18,23]. In parallel, mouse immunization and phage display were also utilized to identify potential therapeutic Abs against SARS-CoV-2 [24]. In order to optimize treatment efficacy, it is desirable to develop cocktails of nAbs that can simultaneously bind different sites of the RBD and synergistically neutralize SARS-CoV-2 [14,25,26].

Here, we describe the generation of a panel of monoclonal antibodies (mAbs) using hybridoma screening. These mAbs potently neutralized SARS-CoV-2 *in vitro* by targeting the RBD of SARS-CoV-2 S protein with high affinity. The 12 most effective neutralizing chimeric Abs (chAbs) exhibited potent neutralizing capability to reduce 50% of plaque counts in the plaque reduction neutralization test (PRNT), yielding the PRNT_50_ values at low nM in an authentic SARS-CoV-2 plaque assay. Site-directed mutagenesis within the RBD identified three key residues within the receptor binding motif (RBM) required for neutralizing activities of these mAbs. Cryo-electron microscopy (cryo-EM) revealed atomic details of the structural epitopes of two representative chAbs, which could potentially be used in a cocktail therapy. The prophylactic and therapeutic potentials of these antibodies and their combination were confirmed in SARS-CoV-2 mouse and hamster infection models, wherein injection of the therapeutic mAb cocktail markedly reduced the virus titers, underscoring their potential to be used in prevention and treatment of COVID-19. Moreover, a cocktail of therapeutic chAbs targeting separate epitopes on the RBM of SARS-CoV-2 spike protein may increase therapeutic efficacy and decrease the potential for virus escape mutants, providing additional benefit to tackle the emergence of new variants that harbor multiple mutations within the S protein.

## Results

### Generation and characterization of anti-SARS-CoV-2 RBD chAbs

**Fig 1:**
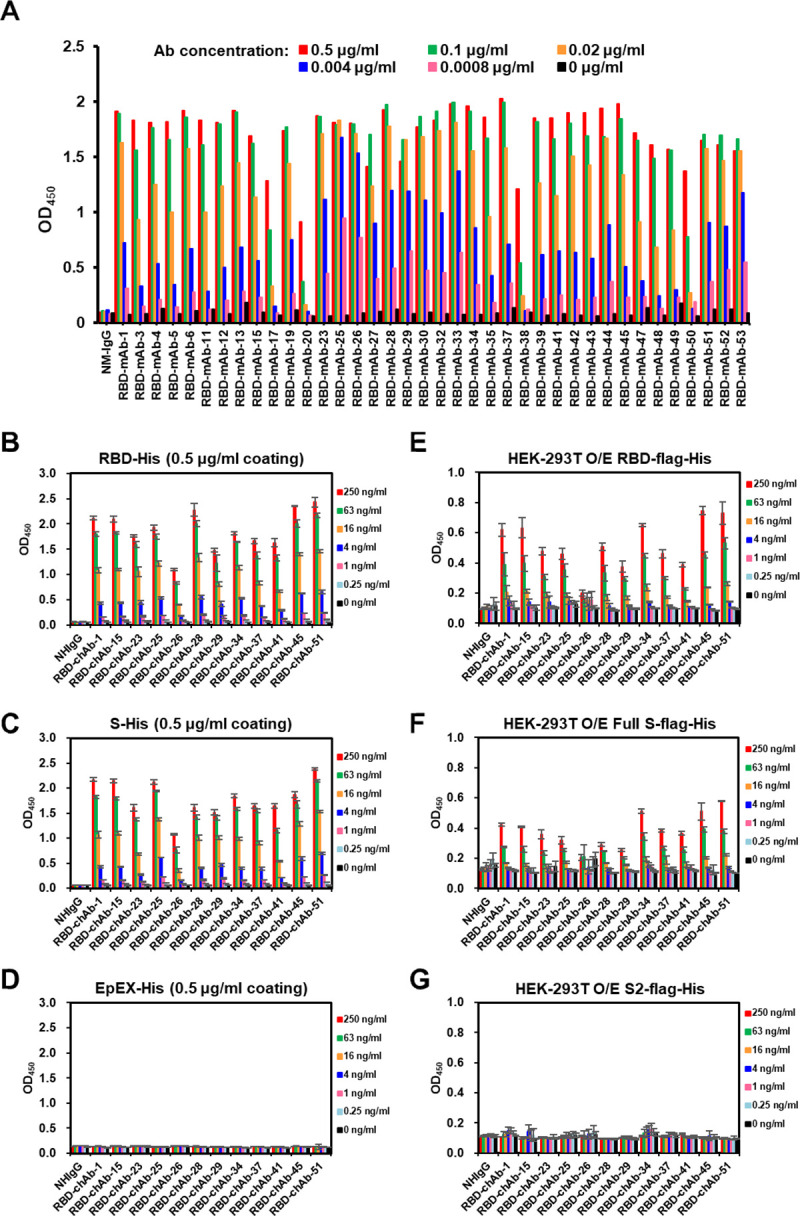
Characterization of mAbs against SARS-CoV-2. A. ELISA-reactivity of anti-RBD mAbs. Each anti-RBD mAb was serially diluted from 0.1 μg/ml to 0.8 ng/ml, then incubated in a RBD-His recombinant protein (0.5 μg/ml)-coated plate in an ELISA. OD_450_, Optical density at 450 nm. B-D. Binding of anti-RBD chAbs was determined by ELISA. SARS-CoV-2 RBD-His or S-His were immobilized on 96-well plates prior to blocking with 1% BSA in PBS and incubated with diluted anti-RBD chAbs at concentrations ranging from 1000 ng/ml to 0.25 ng/ml. Signal was detected (OD) after labeling with Donkey anti-human IgG-HRP secondary antibody. EpEX-His served as a negative control. E-G. Binding of anti-RBD chAbs was assessed by cellular ELISA. HEK-293T cells were transfected with SARS-CoV-2 RBD-flag-His, S2-flag-His or S-flag-His plasmids. A series of dilutions for anti-RBD chAbs were added to the 96-well plates. The ODs were detected with Goat anti-human IgG F(ab’)_2_-HRP secondary antibody. Data information: Except A, each assay was performed in triplicate and all data points are shown, along with the mean ± SD.

BALB/cJ mice were immunized with purified SARS-CoV-2 RBD-Fc protein (S1A Fig) to induce robust serum immune responses (S1B Fig). A total of 38 mAbs were generated, and their binding to the SARS-CoV-2 RBD was determined by ELISA (Fig 1A). We first examined the *in vitro* competition abilities of all RBD-specific hybridoma clones against SARS-CoV-2 using human ACE2-overexpressing 293T cells and flow cytometry (S2A Fig). 17 of the 38 mAbs showed more than 80% inhibition of ACE2 binding to SARS-CoV-2 S RBD. To improve the clinical applicability of these mAbs, the 17 SARS-CoV-2 S RBD-specific mAbs were engineered into human IgG1 chimeric antibodies (chAbs). The V_H_ and V_L_ domains of the neutralizing mAbs from hybridoma cell lines were identified and grafted onto a human IgG1 and kappa backbone to generate 12 chAb clones. The binding of all chAbs to SARS-CoV-2 RBD or S recombinant protein were evaluated by ELISA, and the EpEX-His protein [27] was used as a control protein (Fig 1B–1G). RBD-chAb-28, -45, and -51 exhibited the highest binding signals for recombinant RBD (Fig 1B). RBD-chAb-51 was the most potent, in terms of antigen binding (Fig 1C). HEK-293 cells that overexpress SARS-CoV-2 S RBD on the cell surface were used to evaluate chAb binding. In this context, the binding activities of RBD-chAb-1, -15, -34, -45, and -51 were approximately two-fold weaker compared to the signals to purified protein (Fig 1E–1G). Expression of full-length S protein on the cell surface further confirmed the binding of these chAbs (Fig 1F). As a negative control, HEK-293 cells expressing SARS-CoV-2 S2 domain on the cell surface were not recognized by the chAbs (Fig 1G). To assess the possible neutralization abilities for all 12 chAbs, we performed *in vitro* neutralization studies by PRNT. All tested RBD-specific chAbs, but except RBD-chAb-26 could effectively block authentic SARS-CoV-2 infection of Vero E6 cells (S2B Fig). Notably, the top six most effective RBD-chAbs, namely RBD-chAb-1, -15, -25, -28, -45, and -51 showed PRNT_50_ values of less than 30 ng/ml (S2B Fig).

These six chAbs were highly specific to SARS-CoV-2 S protein. None were found to cross-react with the S proteins of the other six human CoVs, namely SARS-CoV, MERS-CoV, hCoV-OC43, hCoV-HKU1, hCoV-NL63, and hCoV-229E, with the sole exception of RBD-chAb-15, which exhibited partial cross-reactivity to the S1 domain of SARS-CoV S protein (S3A Fig). Additional assessments of antibody specificity (lack of cross-reactivity with whole organs) were performed by staining the FDA human normal organ tissue array. The appropriate amounts of antibodies required for immunocytochemistry staining were assessed using RBD-expressing 293T cells as a reference. All six chAbs showed clear binding to RBD-expressing cells at 1 μg/ml (S3B Fig and Table A in S1 Text). We then used a higher concentration of 5 μg/ml to examine the cross-reactivity of each chAb with a multi-normal tissue array. No tissue cross-reactivity was observed for six major target organs (lungs, liver, spleen, heart, kidney, and larynx) for any of the tested chAbs (S3C Fig and Table A in S1 Text). Further analyses with 27 other human organs, including the cerebrum, cerebellum, adrenal gland, ovary, pancreas, parathyroid gland, hypophysis, testis, thyroid gland, breast, tonsil, thymus, bone marrow, cardiac muscle, esophagus, stomach, small intestine, colon, salivary gland, prostate, endometrium, uterine cervix, skeletal muscle, skin, peripheral nerve, mesothelium, and retina, also did not show cross-reactivity for any of the chAbs (Table A in S1 Text).

### Neutralizing abilities of anti-RBD chAbs

**Fig 2:**
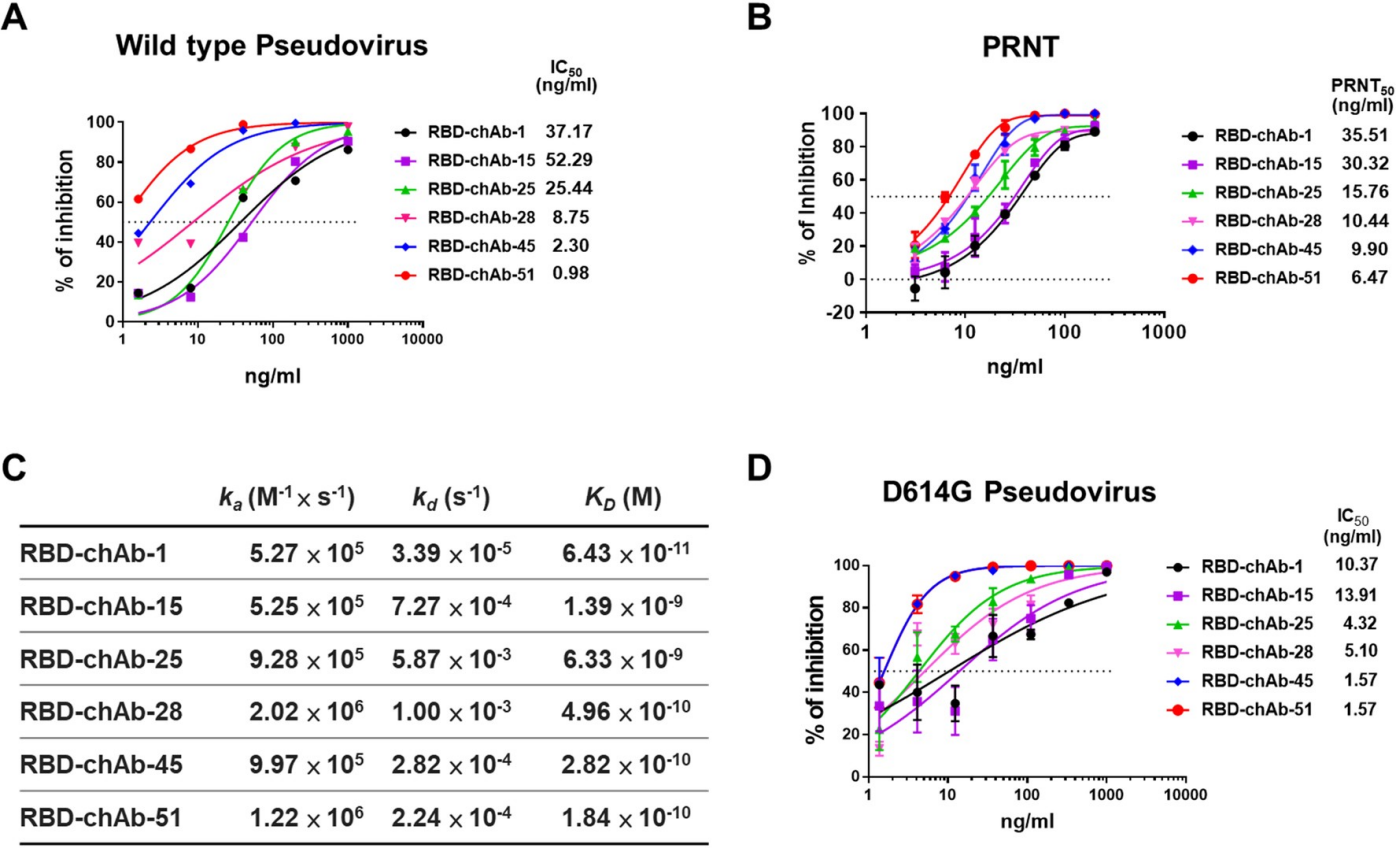
Identification of SARS-CoV-2-neutralizing chAbs. A. Neutralization potency was measured with a pseudotyped virus neutralization assay. Data for each chAb are shown from a representative neutralization experiment. Each assay was performed in triplicate, and all data points represent the mean. B. Neutralizing chAbs inhibiting SARS-CoV-2 infection were assessed by PRNT. ChAbs were serially diluted in PBS and used to block infection of Vero E6 cells with SARS-CoV-2. Virus without chAb served as a control. Plaques formed at each dilution were counted 4 days after virus infection. The PRNT_50_ value was calculated by Prism software. Each assay was performed in duplicate or triplicate and all data points are shown, along with the mean ± SD. C. BLI sensorgrams and kinetics of chAb binding to SARS-CoV-2 RBD. *K*_*D*_ is the affinity constant calculated using a 1:1 binding model. *k*_*a*_, association constant; *k*_*d*_, dissociation constant. D. Neutralization assay of D614G mutant SARS-CoV2 pseudovirus with chimeric anti-RBD antibodies. Each assay was performed in triplicate; data points represent the mean.

According to the pseudovirus neutralization assay, RBD-chAb-28, -45 and -51 exhibited very low IC_50_ values of 8.75, 2.30, and 0.98 ng/ml, respectively (Fig 2A). Additionally, the PRNT showed that all six RBD-chAbs potently neutralized authentic SARS-CoV-2 infection of Vero E6 cells. RBD-chAb-28, -45, and -51 showed the most potent neutralization activities, with PRNT_50_ values of 10.44, 9.90, and 6.47 ng/ml, respectively (Figs 2B and S4). According to surface plasmon resonance measurements, the dissociation constants (*K*_*D*_) of the six chAbs ranged between 64.3 pM and 6.33 nM (Figs 2C and S5). Importantly, the *K*_*D*_ values of RBD-chAb-28, -45, and -51 were less than 500 pM. In light of the global dominance of the D614G variant, we tested the neutralization activity against a pseudovirus of the SARS-CoV-2 D614G mutant, and showed that our six chimeric antibodies retained high neutralizing activities, especially RBD-chAb-25, 45, and -51 (Fig 2D).

### Identification of neutralizing epitopes in the RBD

**Fig 3:**
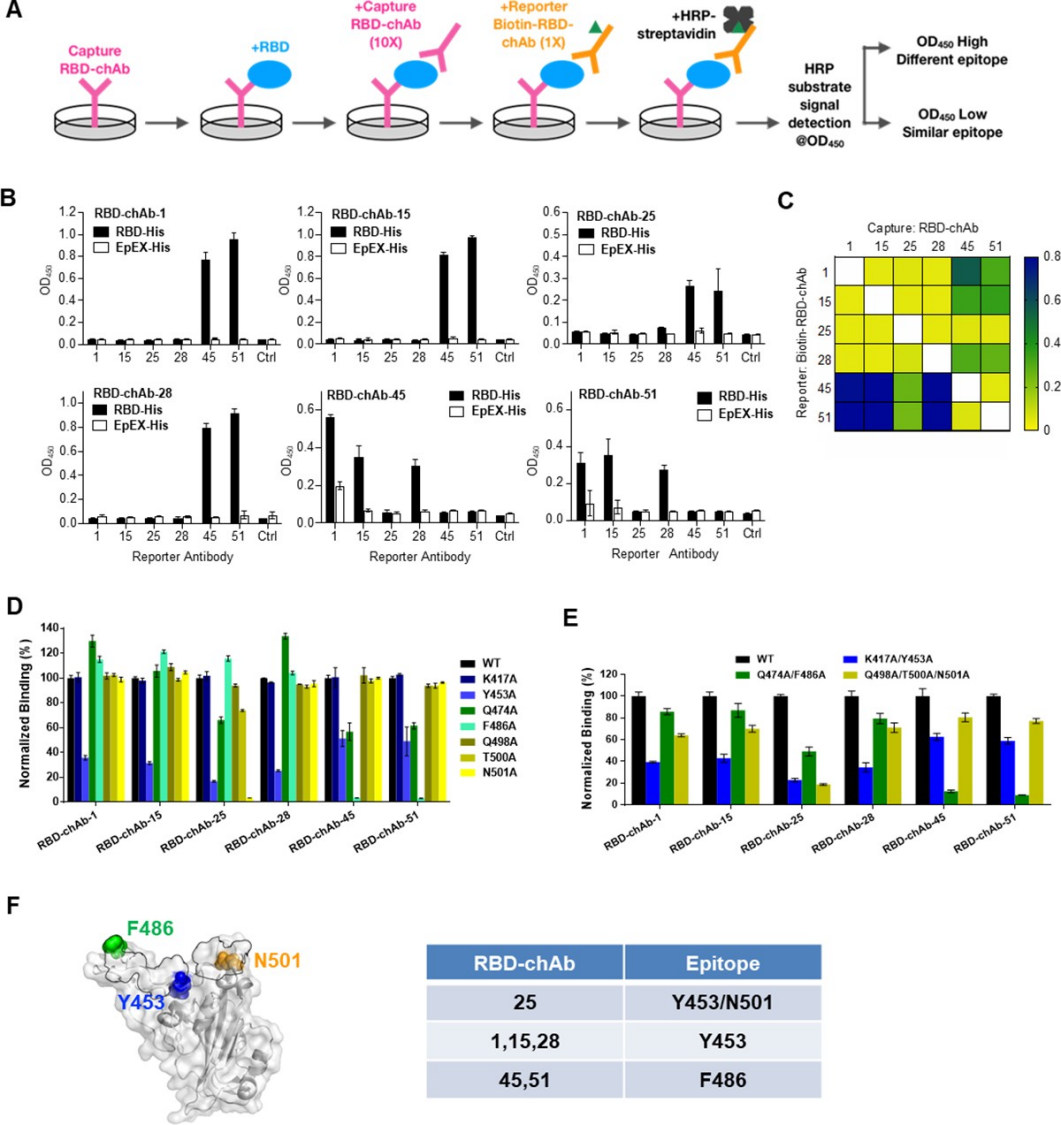
Epitope competitive inhibition and mapping of RBD complex formation for SARS-CoV-2-neutralizing chAbs. A. Schematic illustration of the experimental design of the epitope competition-binding assay. First, RBD-His was captured by RBD-neutralizing chAbs on a 96-well plate. Second, 10-fold capture antibody was added to saturate the RBD. Third, biotinylated RBD-chAb was added to compete with capture RBD-chAb. Finally, HRP-conjugated streptavidin was added to bind the biotinylated reporter RBD-chAb, and competitive binding was detected by optical density at 450 nm (OD_450_). B. Results of triplicate epitope competition-binding assays for RBD-chAb-1, -15, -25, -28, -45, and -51 are shown. EpEX-His served as a negative control (Crtl: without biotin-RBD-chAb). All data points are shown, along with the mean ± SD. C. Heatmap of the epitope competition-binding assay results. The detected OD_450_ values are colored according to the scale bar shown in the right. D-E. Epitope mapping of RBD-neutralizing antibodies by mutagenesis. Normalized binding to RBD alanine variants by RBD-chAbs with respect to that of wild type (WT) based on ELISA. The human 293T cells were transiently transfected with wild-type or mutant RBD plasmids with combinatorial alanine mutations. The binding of RBD-chAbs to the RBD mutants was examined by cellular ELISA. F. Structural mapping of key residues on the RBD responsible for the recognition by RBD-chAbs. The crystal structure of SARS-CoV-2 S RBD in complex with ACE2 (PDB entry: 6M0J) is shown on the white/grey surface. Regions in the RBD within 4 Å of any atoms of ACE2 (defined as RBM) are outlined in black. The positions of the key residues, Y453, F486 and N501, are indicated. Data information: All experiments were performed in triplicate, with the standard deviations shown in error bars.

An ELISA-based competition-binding assay was performed for the six most potent chAbs to examine whether they share overlapping epitopes (Fig 3A–3C). The results suggested overlapping epitopes exist for RBD-chAb-1, 15 and -28; a similar finding was observed for RBD-chAb-45 and -51. Notably, the epitope for RBD-chAb-25 appears to partially overlap with that of RBD-chAb-1, 15 and -28 (Fig 3B–3F). We therefore classified the six chAbs into three distinct groups, each of which recognized a unique epitope on the RBD (Fig 3B and 3C). Structural analysis of ACE2 in complex with SARS-CoV-2 S RBD indicated that K417, Y453, Q474, F486, Q498, T500, and N501 within the RBD make direct contacts with ACE2 forming part of the RBM [28]. These residues are categorized into three clusters, namely Q498, T500 and N501 at the proximal end of the RBM, K417 and Y453 in the middle of RBM, and Q474 and F486 at the distal end of the RBM [28]. To dissect the contributions of these residues to the neutralizing effects of our RBD-chAbs, we carried out alanine scanning of the residues followed by ELISA to assess their impacts on RBD-chAb binding (Fig 3D). The results showed that the singleton mutations at Y453 or N501 significantly decreased the binding signals for RBD-chAb-25, as did the mutation at Y453 for RBD-chAb-28 (Fig 3D). Moreover, RBD-chAb-45 and -51 responded similarly to different singleton mutations, with the F486 mutation being the most disruptive, suggesting that RBD-chAb-45 and -51 bind to the same epitope (Fig 3D). We subsequently generated combinations of singleton mutations and evaluated the effects on RBD-chAb binding. The K417A/Y453A and Q498A/T500A/N501A mutations were found to substantially reduce binding of RBD-chAb-25. The K417A/Y453A mutations had a similar effect on RBD-chAb-28. The Q474A/F486A mutations were more disruptive for RBD-chAb-45 and RBD-chAb-51 (Fig 3E). These results suggest that the epitope residues recognized by RBD-chAb-25 are Y453 and N501, while the epitope residue of RBD-chAb-28 is Y453. Moreover, both RBD-chAb-45 and -51 recognize F486 of the RBD (Fig 3F).

### Cryo-EM analysis of RBD-chAbs in complex with SARS-CoV-2 S protein

**Fig 4:**
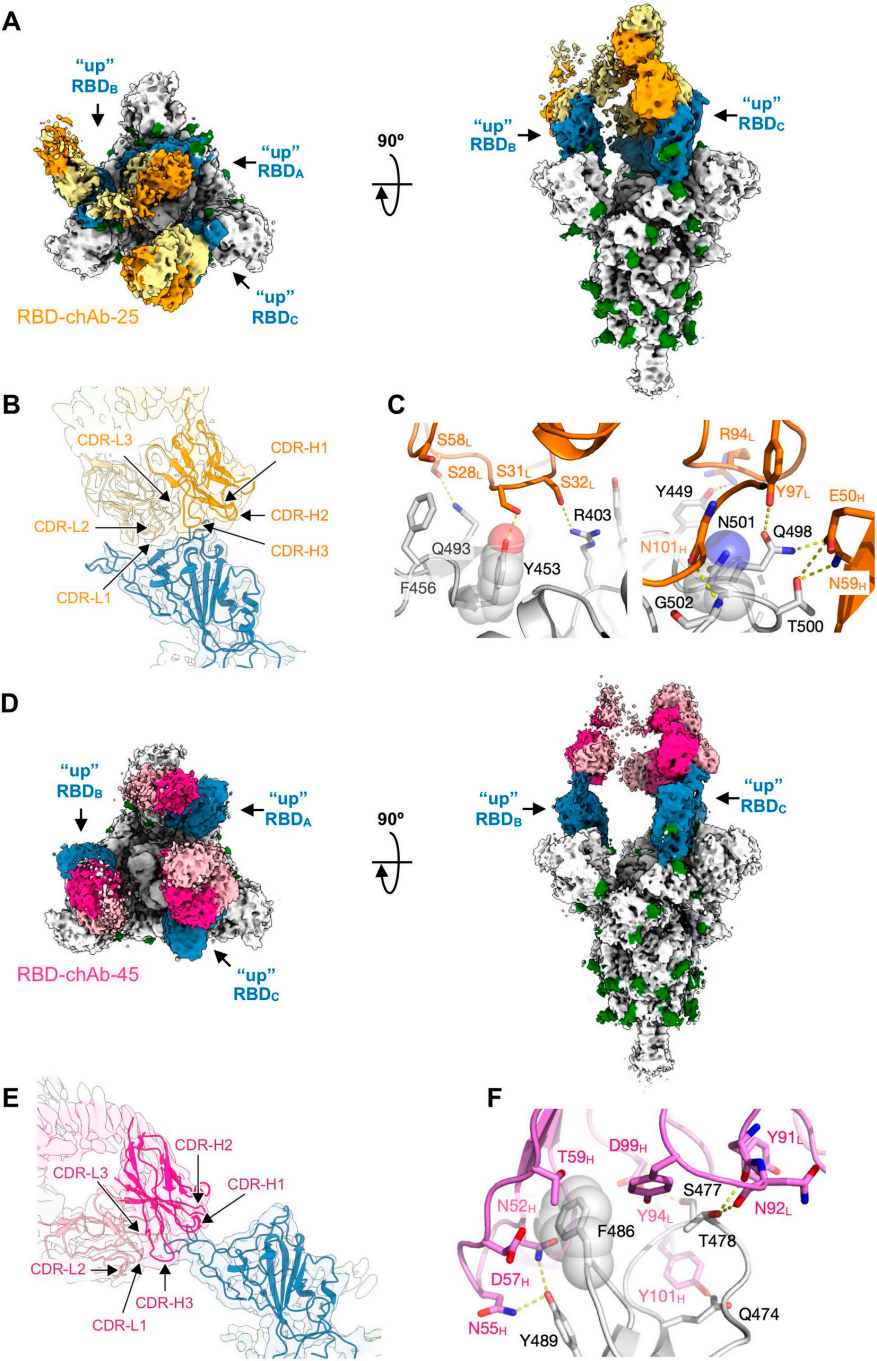
Cryo-EM structure of RBD-chAb-25 and -45 in complex with SARS-CoV-2 S protein. A, D. Orthogonal views of the EM maps of RBD-chAb-25 (A) and -45 (D) in complex with SARS-CoV-2 S protein. The three RBDs are colored in blue, and the glycans are colored in green. The heavy chain and light chain of RBD-chAb-25 are colored in orange and pale yellow, respectively. The HC and LC of RBD-chAb-45 are colored in fuchsia and pink, respectively. B, F. Expanded views of RBD-chAb-25 (B) and -45 (E) in complex with SARS-CoV-2 S protein. The EM maps are shown as transparent surfaces with the atomic models shown as schematic representations. The positions of the CDR loops are indicated. C, E. Atomic details of the molecular recognition of RBD-chAb-25 (C) and -45 (F). The side-chains of the key recognition residues on the RBD, Y453, F486 and N501, are indicated by transparent spheres. Intermolecular hydrogen bonds are shown as dashed yellow lines. Data information: The identities of individual residues are indicated and colored accordingly.

To reveal the structural basis of how the distinct classes of the RBD-chAbs recognize the SARS-CoV-2 S protein, we determined the cryo-EM structures of RBD-chAb-25 and -45 in complex with the ectodomain of the SARS-CoV-2 S protein (Figs 4, and S6 and S7). In both cases, the chAbs bound to the SARS-CoV-2 S protein in a 3:3 stoichiometry, indicated by the three distinct EM densities protruding from the three RBDs, which were all in the open conformation (Fig 4). The overall nominal resolutions of the S-chAb complexes were between 3.6 and 3.5 Å (S-chAb-25 and S-chAb-45 complex, respectively, Table B in S1 Text). Focused refinement of the cryo-EM maps by masking the Fab and RBD to yield a better definition of the binding interface, thus enabling *de novo* model building of the Fabs and the S protein to define the atomic details of the epitopes of individual RBD-chAbs (Materials and Methods).

Detailed structural analysis showed that RBD-chAb-25 bound to the RBD via an extensive intermolecular hydrogen bond network around Y453 and N501 (Figs 4C and S8A). Specifically, Y453, Q493 and R403 of the RBD were hydrogen bonded to S31_L_, S28_L_ and S32_L_ of RBD-chAb-25 (subscript L denotes the light chain), respectively. Additionally, G502 (adjacent to N501) of the RBD and N101_H_ of RBD-chAb-25 (subscript H denotes the heavy chain) formed a backbone-to-backbone hydrogen bond. A cluster of bipartite hydrogen bonds was also formed at the interface of the light chain (Y97_L_) and heavy chain (E50_H_ and N59_H_) of RBD-chAb-25, with Q498 and T500 of the RBD. The overall binding interfaces between the RBD and the light/heavy chains of RBD-chAb-25 were 511 Å^2^ and 350 Å^2^, respectively. In the case of RBD-chAb-45, the phenyl ring of the key residue F486 on the RBD was encaged by the side chains of Y94_L_, N52_H_, D57_H_, and T59_H_ of RBD-chAb-45, adjacent to a bipartite hydrogen bond between Y489 of the RBD and N52_H_, and N55_H_ of RBD-chAb-45 in close proximity to F486 of the RBD (Figs 4F and S8B). Additionally, T478 of the RBD was hydrogen-bonded to Y91_L_ and N92_L_ of RBD-chAb-45. The overall binding interfaces between the RBD and the light and heavy chains RBD-chAb-45 were 190 Å^2^ and 362 Å^2^, respectively.

**Fig 5:**
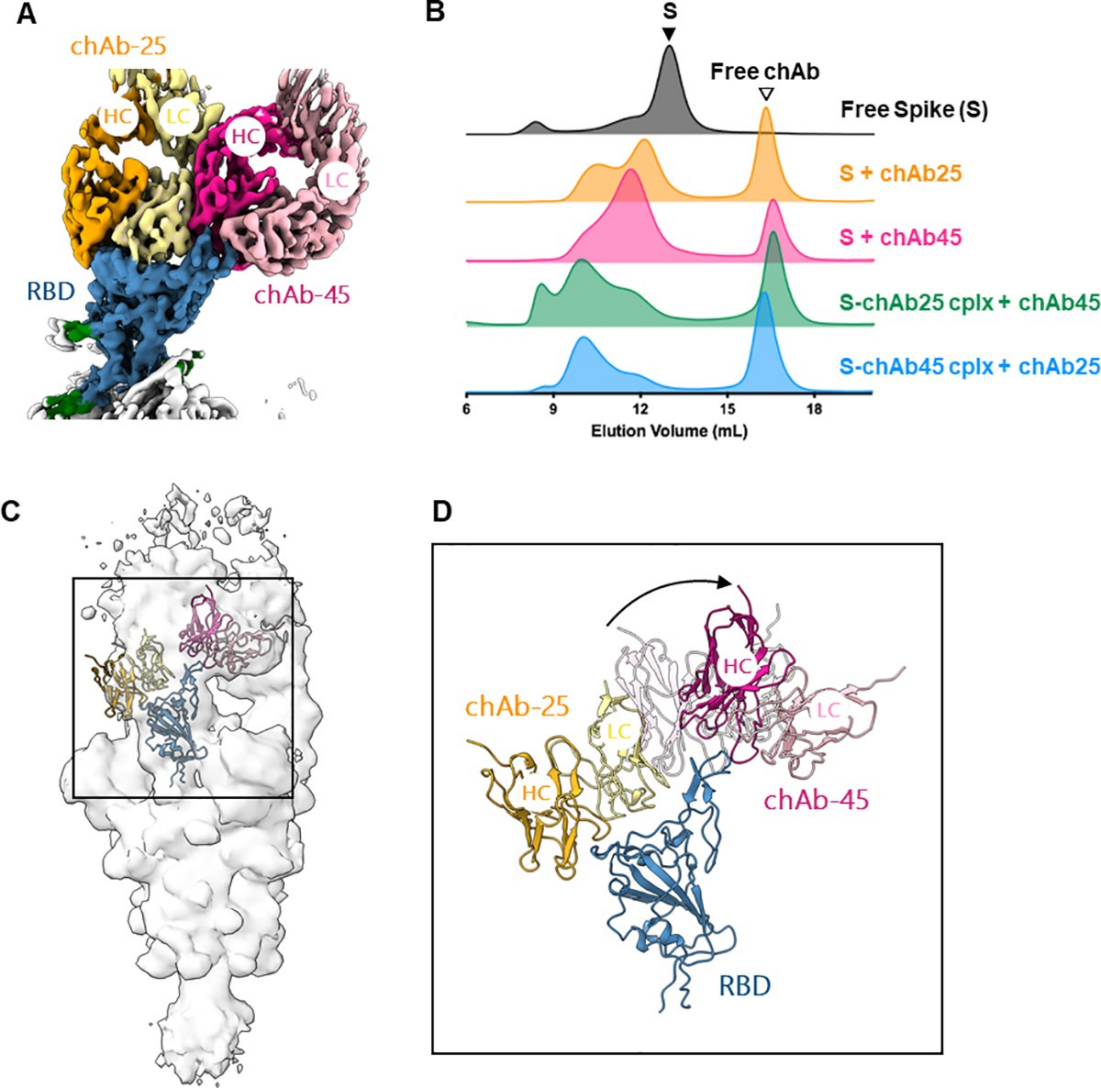
Structural and biophysical evidence of simultaneous binding of two RBD-chAbs to SARS-CoV-2 S protein. A. Overlay of cryo-EM maps of the Fabs of chAb-25 and -45 to the same RBD. The color scheme is the same as that of Fig 4.B. Size-exclusion chromatograms (SEC) of the free S protein (black), RBD-chAb-25-bound S protein (orange), RBD-chAb-45 bound S protein (fuchsia), addition of RBD-chAb-45 to pre-mixed RBD-chAb-25-bound S protein (green), and addition of RBD-chAb-25 to pre-mixed RBD-chAb-45-bound S protein (blue). Excess RBD-chAbs were added to the mixture, resulting in free RBD-chAb elution peaks, indicated by the open triangle. cplx, complex. C. Cryo-EM map of the ternary complex of SARS-CoV-2 S protein in complex with chAb-25 and 45 with the atomic models of the RBD, Fabs of chAb-25 and 45 fit to the EM map of one of the three protomers. The cartoon representations are colored in the same scheme as (A). D. Expanded view of the cryo-EM map-derived structural model of RBD-bound to the Fabs of chAb-25 and 45 corresponding to the boxed region in (C). A rigid body rotation of chAb-45 relative to the singly bound chAb-45 (shown in transparent cartoon) is indicated by an arrow.

Despite some overlaps in the structural epitopes of RBD-chAb-25 and -45, superposition of the two resolved Fab structures onto the same RBD showed few steric clashes between the Fabs, suggesting that the two RBD-chAbs could bind simultaneously to the same RBD (Fig 5A). To verify their simultaneous binding, we mixed RBD-chAb-25 and SARS-CoV-2 S protein and isolated the binary complex by size-exclusion chromatography (SEC), followed by the addition of RBD-chAb-45 for another round of SEC analysis (Fig 5B). A clear shift in the elution volume of the main peak was observed, indicating the formation of a ternary complex, wherein the added RBD-chAb-45 was bound to the complex of RBD-chAb-25 and SARS-CoV-2 S protein, despite the limited space around the three RBDs to accommodate more than three chAbs. The same shift in elution peak was observed when RBD-chAb-25 was added to the complex of RBD-chAb-45 bound SARS-CoV-2 S protein, i.e., in reverse mixing order (Figs 4A, 4D and 5B). These changes in SEC elution profiles provided clear evidence of simultaneous binding of RBD-chAb25 and -45 to SARS-CoV-2 S protein to form a higher order complex.

Evidence of simultaneous binding of two different RBD-chAbs to the same RBD was even more clearly observed when isolated RBD was used to form a quaternary complex with RBD-chAb-25 and -45; better resolved SEC elution profiles could be observed in these experiments (S9 Fig). The addition of RBD-chAb-25 or RBD-chAb-45 resulted a clear shift of the elution volume of the main elution peak, indicating the formation of a stable binary complex between the RBD and the individual nAbs. Similar to the use of a trimeric SARS-CoV-2 S protein, subsequent addition of a second nAb to a pre-formed RBD-nAb complex resulted in further shift of the elution volume to a higher molecular weight, indicating the formation of a ternary complex formed by two different nAbs and the RBD, regardless of the mixing sequence of the nAbs (S9 Fig). Collectively, these SEC analyses of different S protein constructs provided strong evidence of the ability of RBD-chAb-25 and -45 to simultaneously bind to the RBD.

To verify the formation of a ternary complex between RBD-chAb-25 and -45 with the SARS-CoV-2 S protein, we determined the cryo-EM map of the ternary complex that was purified by SEC as shown in Fig 2B (S-chAb25 cplx + chAb45). The resolution of the EM map was limited, in part due to the conformational heterogeneity of the nAbs in complex with the RBD. However, the resolution was sufficient for us to dock the Fabs of RBD-chAb-25 and -45 onto the RBD, which required considerable conformational rearrangements of the relative orientation of the Fab of chAb-45 with respect to the binary S-chAb-45 complex for one of the three RBDs (Fig 5C and 5D). While the cryo-EM map of the Fab of RBD-chAb-45 could be seen at a lower threshold for the other two RBDs, the EM map corresponding to the Fab of RBD-chAb-25 was less visible in the other two RBDs. The lack of EM-density from RBD-chAb-25 could be attributed to either conformational heterogeneity or substoichiometric binding. While the exact binding stoichiometry of the ternary complex remains to be established, our SEC and cryo-EM analyses provided good evidence of the simultaneous binding of RBD-chAb-25 and -45 to the SARS-CoV-2 S RBD, that could serve as the basis for antibody cocktail therapy developments.

### Prophylactic effect of RBD-chAb in SARS-CoV-2-infected mice or hamsters

**Fig 6:**
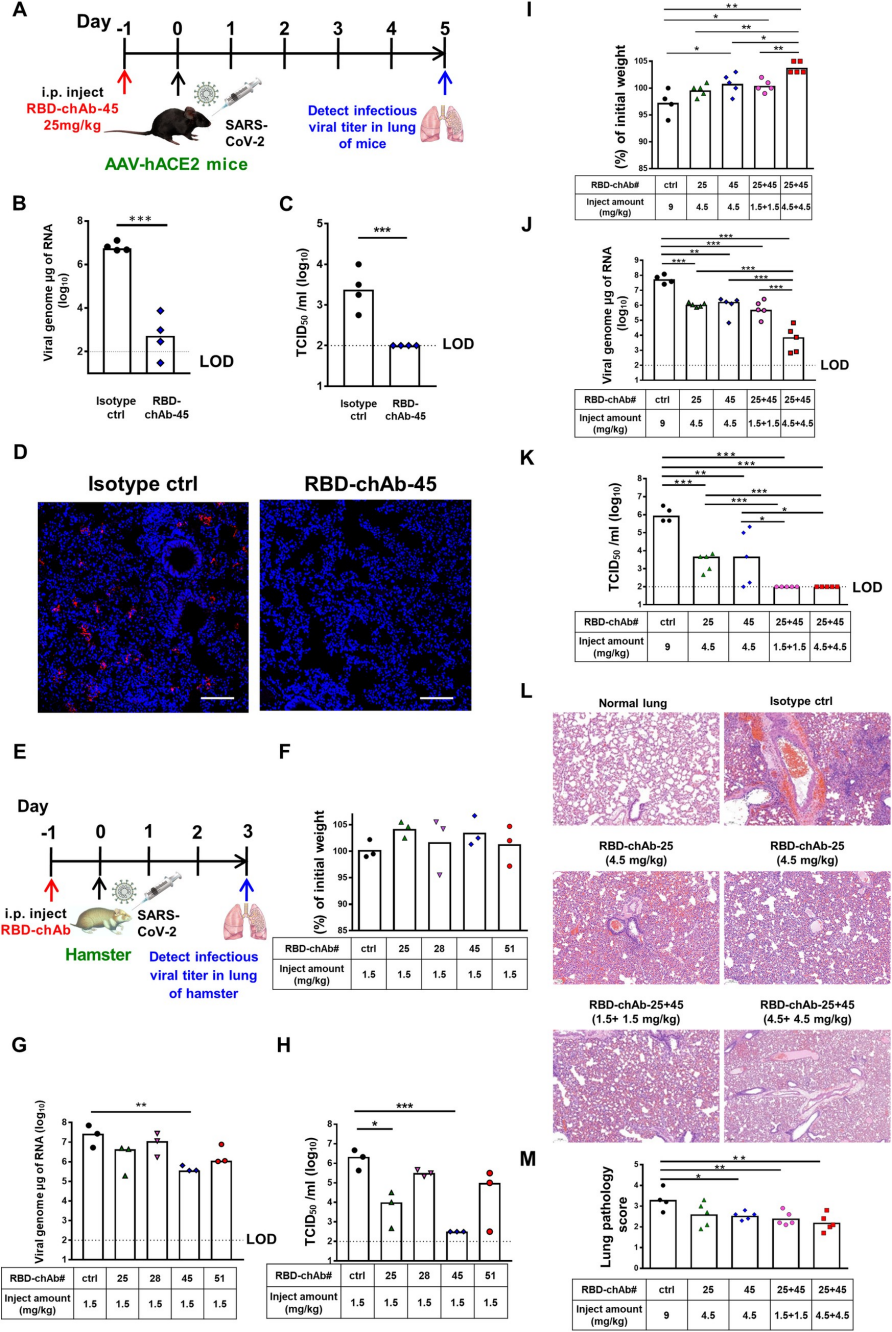
Prophylactic efficacy of neutralizing chAbs against SARS-CoV-2 infection. A. Illustration of the study design for prophylactic efficacy of RBD-chAb-45 against SARS-CoV-2 in AAV-ACE2 mice. One day prior to intranasal challenge of SARS-CoV-2, each group of mice was given a single intraperitoneal dose of 25 mg/kg of RBD-chAb-45 (n = 4), or NHIgG, normal human IgG, as isotype control (n = 4). On day 5 after virus inoculation, lung samples were collected for analysis. B-C. The viral load in the lung of mice treated RBD-chAb-45 was determined by qRT-PCR and median tissue culture infectious dose per ml (TCID_50_/ml) was calculated. D. Viral antigen was detected by anti-SARS-CoV-2 N protein mAb (red) in paraffin embedded lung tissue. Nuclear DNA was stained with DAPI (blue). E. Illustration of the study design for prophylactic efficacy of RBD-chAb against SARS-CoV-2 in hamsters. One day prior to intranasal challenge of SARS-CoV-2, each group of hamster was given a single intraperitoneal injection of RBD-chAbs (n = 3 or 5), or NHIgG as isotype control (n = 3 or 4). On day 3 after virus inoculation, lung samples were collected for analysis. F. The percentage of body weight of hamsters treated single RBD-chAb were compare to the body weight in the day of virus inoculation. G-H. The viral load in the lung of hamsters treated single RBD-chAb was determined by qRT-PCR and median tissue culture infectious dose per ml (TCID_50_/ml) was calculated. I. The percentage of body weight of hamsters treated cocktail RBD-chAbs were compare to the body weight in the day of virus inoculation. J-K. The viral load in the lung of hamsters treated cocktail RBD-chAbs was determined by qRT-PCR and median tissue culture infectious dose per ml (TCID_50_/ml) was calculated. L-M. The pathologic changes in the lung were assessed by immunohistochemistry. Data information: All data points are shown, along with the median. * *p* < 0.05, *** *p* < 0.001, as determined by Student’s *t*-test. ctrl, isotype control. i.p., intraperitoneal. LOD, limit of detection, 1 × 10^2^ TCID_50_/ml. Scale bars, 100 μm. The lung pathology score definition is according to Table C in S1 Text.

To assess the *in vivo* prophylactic potency for SARS-CoV-2 infection, we selected RBD-chAb-45 for the first evaluation based on its high neutralization capacity. An adeno-associated virus (AAV)-mediated human ACE2-expressing (AAV-hACE2) mouse model was administered a single shot of 25 mg/kg antibody one day before SARS-CoV-2 infection (Fig 6A–6D). The virus titer was significantly lower than controls and the plaque-forming units were undetectable in the treatment group at 5 days post-infection of SARS-CoV-2 (Fig 6B and 6C). This result was confirmed by immunohistochemical staining of tissues from treated animals at 5 days post-infection (Fig 6D), confirming the potent *in vivo* neutralization activity of RBD-chAb-45 against SARS-CoV-2.

**Fig 7:**
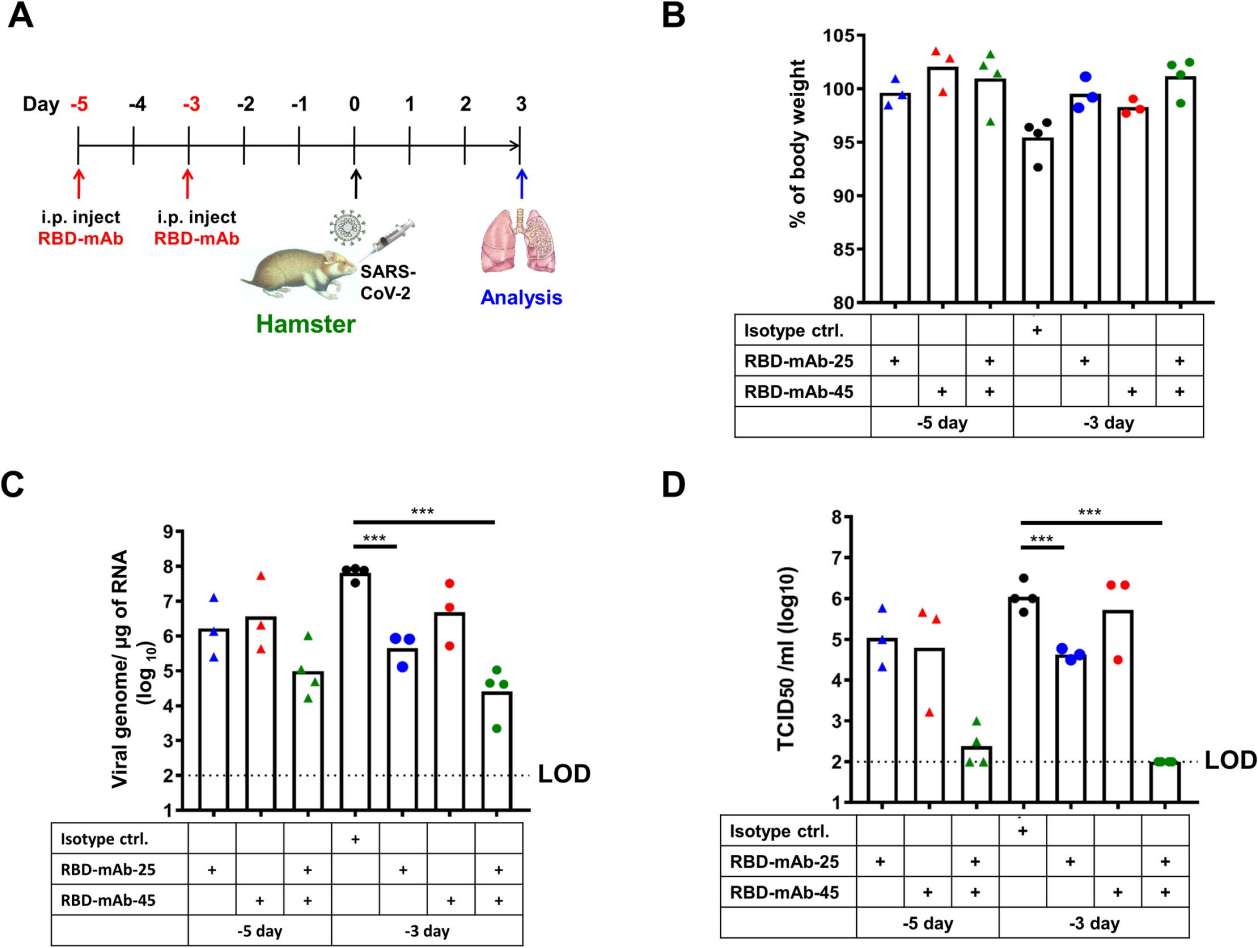
Prophylactic efficacy of mouse monoclonal neutralizing antibody cocktail against SARAS-CoV-2 infection in hamsters. A. Illustration of the study design for prophylactic efficacy of monoclonal mouse antibodies, against SARS-CoV-2 in hamster. Three or five days prior to intranasal challenge of SARS-CoV-2, each group of hamster was given a single intraperitoneal dose of 1.5 mg/kg of RBD-mAb-25 (n = 3), RBD-mAb-45 (n = 3), or 3 mg/kg of RBD-mAb-25 combined RBD-mAb-45 (n = 4), 3 mg/kg of NMIgG as isotype control (n = 4). On day 3 after virus inoculation, body weights were recorded and lung samples were collected for analysis. B. The percentage of body weight were compare to the body weight in the day of virus inoculation. C. The viral load in the lung was determined by qRT-PCR. D. The infectious viral load in the lung was determined by median tissue culture infectious dose per ml (TCID_50_/ml) was calculated. Data information: All data points are shown, along with the median. *** *p* < 0.001, as determined by Student’s *t*-test. ctrl, isotype control. i.p., intraperitoneal. LOD, limit of detection, 1 × 10^2^ TCID_50_/ml.

Next, we used a hamster model to mimic virus transmission in mild human SARS-CoV-2 infection [29]. We administered a single intraperitoneal injection of low-dose RBD-chAb-25, -28, -45 and -51 at 1.5 mg/kg one day prior to SARS-CoV-2 infection (Fig 6E–6H). The virus titer was determined from the lung tissue of each hamster at the third day after infection. Although no body weights were changed (Fig 6F) and only RBD-chAb-45 caused a statistically significant decrease in the level of virus RNA measured by RT-qPCR (Fig 6G), the TCID_50_ values were decreased in all chAb-treated groups, and the effect was especially significant in the RBD-chAb-45-treated group at the third day post-infection compared to the control group (Fig 6H). We further assessed the efficacy of the cocktail of the two best RBD-chAbs (RBD-chAb-25 and -45) to hamsters (Fig 6I–6K). A single intraperitoneal injection of 1.5 or 4.5 mg/kg both RBD-chAb or 4.5 mg/kg single RBD-chAb one day prior to SARS-CoV-2 infection conferred dramatic protection, according to the infectious SARS-CoV-2 titers at the third day post infection. Body weights were not changed in the group injected with 1.5 mg/kg antibody and may have even been slightly increased in the group receiving 4.5 mg/kg antibody (Fig 6I). Importantly, the virus RNA and TCID_50_ values were decreased drastically in groups that received combinations of RBD-chAb-25 and -45 (1.5 or 4.5 mg/kg of each antibody, 3 or 9 mg/kg of total antibody at 3 days post-infection (Fig 6J and 6K). As the lower dose of neutralizing antibodies may induce antibody-dependent enhancement infection in SARS-CoV-2 infected hamsters [30], we tested 1.5 or 4.5 mg/kg of each RBD-mAb-25, -45, or the combination treated at three or five days prior to intranasal challenge of SARS-CoV-2 in hamsters (Fig 7A). No body weight loss was observed at the third day after virus challenge (Fig 7B), and significant reduction of viral load was seen (Fig 7C and 7D). Notably, when using the low dose of these two antibodies (1.5 mg/kg) or their combination (3.0 mg/kg), we did not find that any of the neutralizing antibodies enhance disease. In groups receiving a combination of RBD-chAb-25 and -45, the neutralizing activity (TCID_50_ values) exhibited a synergistic effect compared to those receiving single treatments of RBD-chAb-25 or -45 (Figs 6K and 7D).

### Therapeutic effect of RBD-chAb cocktail in SARS-CoV-2-infected mice or hamsters

**Fig 8:**
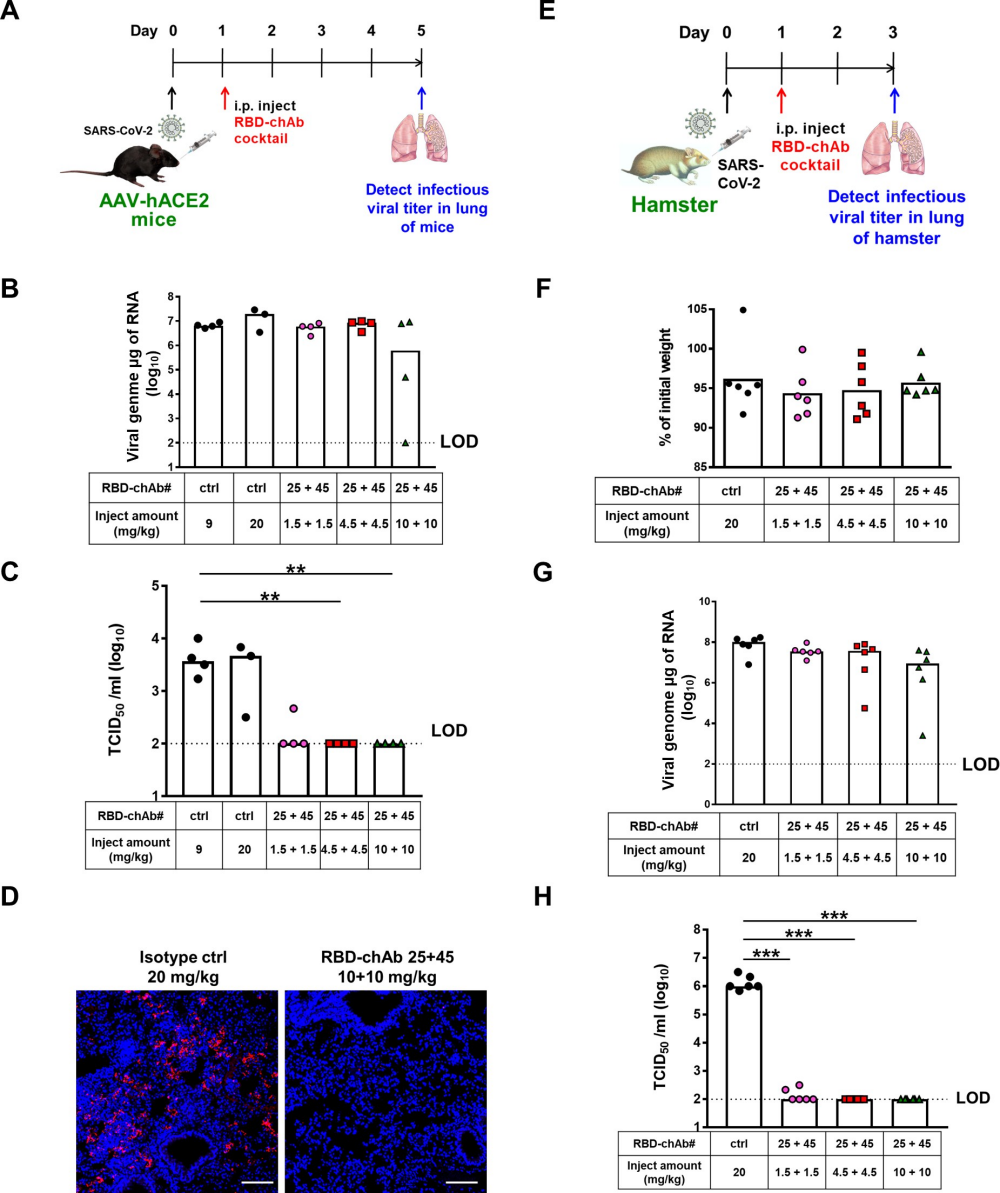
Therapeutic efficacy of neutralizing chAbs against SARS-CoV-2 infection. A. Illustration of the study design for therapeutic efficacy of cocktail RBD-chAbs against SARS-CoV-2 in AAV-hACE2 mice. One day after to intranasal challenge of SARS-CoV-2, each group of mice was given a single intraperitoneal dose of 1.5, or 4.5, or 10 mg/kg of RBD-25 + 45 (n = 4) or 9 or 20 mg/kg NHIgG, normal human IgG, as isotype control (n = 7). B-C. On day 5 after virus inoculation, the viral load in the lung of mice treated cocktail RBD-chAbs was determined by qRT-PCR and median tissue culture infectious dose per ml (TCID_50_/ml). D. Viral antigen was detected by anti-SARS-CoV-2 N protein mAb (red) in paraffin embedded lung tissue. Nuclear DNA was stained with DAPI (blue). E. Illustration of the study design for therapeutic efficacy of cocktail RBD-chAbs against SARS-CoV-2 in hamster. One day after to intranasal challenge of SARS-CoV-2, each group of hamsters was given a single intraperitoneal dose of 1.5, or 4.5, or 10 mg/kg of each RBD-chAb (n = 6), or 20 mg/kg NHIgG as isotype control (n = 6). F. The percentage of body weight of hamsters treated cocktail RBD-chAbs were compare to the body weight in the day of virus inoculation. G-H. On day 5 after virus inoculation, the viral load in the lung of hamsters treated cocktail RBD-chAbs was determined by qRT-PCR and median tissue culture infectious dose per ml (TCID_50_/ml). Data information: All data points are shown, along with the median. ** *p* < 0.01, *** *p* < 0.001, as determined by Student’s *t*-test. ctrl, isotype control. i.p., intraperitoneal. LOD, limit of detection, 1 × 10^2^ TCID_50_/ml. Scale bars, 100 μm.

We next tested the effect of treating animals with the antibody cocktail post SARS-CoV-2 infection. We treated the AAV-hACE2 mouse model with combinations of 1.5, 4.5, or 10 mg/kg of each RBD-chAb-25 and -45 at one day post-intranasal SARS-CoV-2 inoculation (Fig 8A). Although the viral genome RNA could be detected, the infectious SARS-CoV-2 titers were close to the limit of detection (LOD, 1 × 10^2^ TCID_50_/ml) for all mice of the RBD-chAb cocktail-treated groups at 5 days post-infection (Fig 8B and 8C). The viral antigen contents were also assessed in lung tissue of mice treated RBD-chAb cocktail (10 mg/kg of each antibody) using immunohistological assays, and no or very few viral antigens were detected (Fig 8D). Next, we tested the therapeutic effects in the hamster model (Fig 8E–8H). However, the viral genome RNA could still be detected at the end of the experiment and the body weight of the hamsters showed a slight loss, similar to the control group (Fig 8F and 8G). As expected, the combination of RBD-chAb-25 and -45 exhibited the same pronounced therapeutic effect when administered 1 day post-intranasal SARS-CoV-2 inoculation in hamsters or AAV-hACE2 mice (Fig 8H). Collectively, our data demonstrated an additive neutralizing effect of the RBD-chAb cocktails of RBD-chAb-25 and -45, which acted as prophylactic and therapeutic agents for SARS-CoV-2 infection in both mice and hamsters.

## Discussion

Effective nAbs are highly sought after for the fight against the COVID-19 pandemic because of their abilities to slow down the spread of the virus, and to provide timely treatments for the critically ill. Here we reported the development of a panel of potent chAbs that target distinct structural epitopes within the RBD of SARS-CoV-2 S protein. These chAbs effectively neutralized SARS-CoV-2 in cell cultures with PRNT_50_ values down to 6 ng/ml (Fig 2). We defined three distinct classes of structural epitopes for these RBD-chAbs using a site-directed mutagenesis approach (Fig 3); these classes were further elucidated in atomic detail by cryo-EM structural analyses to guide the design of cocktail therapy against SARS-CoV-2 (Fig 4). The ability of RBD-chAb-25 and -45 to simultaneously bind to the RBD of SARS-CoV-2 S protein was confirmed by SEC (Fig 5). The prophylactic and therapeutic potentials of the cocktail therapy were verified using SARS-CoV-2-infected mouse and hamster animal models (Figs 6–8).

Our *in vitro* and *in vivo* evidence demonstrated high potencies of the identified RBD-chAbs in neutralizing SARS-CoV-2. Indeed, the PRNT_50_ values of our top three neutralizing mAbs, namely RBD-chAb-28 (10.44 ng/ml), -45 (9.90 ng/ml), and -51 (6.47 ng/ml), were better than previously reported nAbs, such as 47D11, BD-368-2, P2B-2F6 and P2B-1F11 [12,13,17], and the PRNT_50_ values of our antibodies were comparable to those for BD-368, REGN-COV2, and CV07-250 [12,23,31]. In particular, RBD-chAb-45 and -51 represent the best in class nAb, in terms of effective dosage for reducing viral RNA in animal models [12,14,16,18,32–34].

Using cryo-EM, we revealed the atomic details of RBD-chAb-25 and -45 binding to the RBD of SARS-CoV-2 S protein (Fig 4). The structural epitopes are generally hydrophilic, and the intermolecular interactions mostly involve hydrogen bonding. Additionally, F486 of the RBD is sandwiched by a T-shaped edge-to-face π-π interaction with Y94_L_ and a CH-π interaction with T59_H_ of RBD-chAb-45 (Fig 4F). This unique binding motif may help stabilize the complex formation despite its relatively small binding interface compared to that of RBD-chAb-25 (Fig 4B and 4E). Of all reported structures for SARS-CoV-2 S protein in complex with antibodies and nanobodies, only six showed all three RBDs in an upward, open conformation [12,14,15,35–45]. By lifting the RBD upward, more binding surface is made available to other nAbs (Fig 4A and 4D). Indeed, molecular modeling of RBD-chAb-25 and -45 in complex with the RBD suggested the possibility that these two Abs could bind simultaneously to the same RBD by occupying two distinct structural epitopes (Fig 5A), a finding which was subsequently confirmed by SEC analyses (Figs 5B and S9). As the collective contributions to the binding interface of RBD-chAb-25 and -45 essentially cover the entire RBM of ACE2 (Fig 3F), the combined use of these two Abs is expected to exhibit strong synergy in neutralizing SARS-CoV-2; this synergistic neutralization was confirmed by our *in vivo* animal model studies (Figs 6K and 7D).

According to the reported epitopes of COVID-19 nAbs, there exist three types of SARS-CoV-2 S protein binding modes: (1) direct binding to the RBM, (2) binding to the RBD outside the RBM [14,15,31,46,47], and (3) binding to the S protein outside the RBD while still exhibiting neutralizing activity [14,46]. Combining multiple nAbs with non-competing epitopes has been demonstrated to synergistically neutralize virus infection [18]. Here, we showed that a cocktail of RBD-chAb-25 and -45 also exhibits synergetic neutralizing ability, and this combination is likely to retain therapeutic potential for SARS-CoV-2 mutants.

Despite the rapid development of multiple nAbs against SARS-CoV-2, mutations in the S protein can potentially lead to drug resistance. Since the release of the first SARS-CoV-2 complete sequence [48], 28811 point mutations have been identified in the SARS-CoV-2 genome [49]. Non-silent mutations in specific residues may severely disrupt nAb epitopes or enhance viral infection. Among these mutants, the D614G point mutation is the most prevalent [50,51]. This point mutation in the S1 domain (not in the RBD) is frequently recognized by nAbs, and it leads to a more stable S protein and higher virus infectivity [51–53]. In addition to the D614G mutant, more transmissible SARS-CoV-2 variants have been reported [54]. While some mutations outside the RBD site were found to escape antibody binding [55], all six of our potent antibodies retained high binding signals when tested with S protein variants harboring some of the most common mutations on the GISAD sequencing database for COVID-19 on December, 2020 (S10A Fig). Residue N501 within the RBM is highly mutable in various infectious SARS-CoV-2 strains, including the recently emerged United Kingdom variant B.1.1.7 and the South African variant B.1.351, which are more infectious than the original strain [56]. We continue to track the binding of these six antibodies to other common SARS-CoV-2 mutant variants, including N501Y, using cellular ELISA (S10B Fig). Nearly all of the antibodies retain the ability to recognize most common mutation variants, with only RBD-chAb-25 showing poor binding to the N501Y mutant (S10B Fig).

Three major mutations of concern within the RBD of SARS-CoV-2 (i.e., N501Y, K417N/K417T and E484K) are present in the highly transmissible B.1.1.7 (United Kingdom), B.1.351 (South Africa) and P.1 (Brazil) variants, and were recently reported to disrupt binding by several prominent nAbs, including REGEN10933, 2–15, LY-Cov555, and CT-P59 [57]. We have tested the neutralization activities of our antibodies against these mutant RBD recombinant proteins using ELISA and pseudotype viruses for B.1.1.7 and B.1.351 (S10C–S10F Fig). Fortunately, RBD-chAb-45 and -51, which share almost the same epitope, retained high binding ability for all three major variants of concern. Although RBD-chAb-25 lost its binding ability for the N501Y mutant form of RBD and B.1.1.7 or B.1.351 pseudoviruses, it still retained the ability to recognize K417N and E484K mutant RBD proteins (S10C Fig). Furthermore, the antibody cocktail, RBD-chAb-25 and -45, was tested against D614G, B.1.1.7, and B.1.351 pseudoviruses. The cocktail showed IC_50_ values close to those of RBD-chAb-45 only, meaning that the effect was not diminished due to the loss neutralization ability for RBD-chAb-25 (S10F Fig). Although mutations of N501 could perturb the binding of RBD-chAb-25 to the RBD of SARS-CoV-2 (Fig 4C), none of the reported mutations within the RBD overlaps with the epitope of RBD-chAb-45 (Fig 4F). In addition, the E484K mutation is not located within the binding surfaces of RBD-chAb-25 and RBD-chAb-45 according to our cryo-EM analysis (S11 Fig). Although the K417N mutation is located within the binding epitope of RBD-chAb25, it is at the edge of the binding surface (S11 Fig), and RBD-chAb25 still retains the ability to bind the RBD (S10C Fig). These findings suggest that the cocktail of RBD-chAb-25 and -45 might be effective at overcoming drug resistance due to escape mutations. This is similar to REGEN10987, which retains neutralizing activity for SARS-CoV-2 variants B.1.1.7 and B.1.351 [57]. Interestingly, we have identified the cryo-EM structure of RBD-chAb-15 in complex with SARS-CoV-2 S protein and found the combination of RBD-chAb-15 and -45 show a synergistic effect toward B.1.1.7 in the pseudovirus neutralization assay [58]. Therefore, our six chimeric antibodies can be used strategically to create cocktail therapies against multiple SARS-CoV-2 mutant strains.

Synergistic effects of antibody cocktail therapies have been reported, which paved the way for the anti-SARS-CoV-2 S protein antibodies, REGN 10987 and REGN 10933, to enter clinical trials, even without animal experiments [25]. Additionally, Liu and co-workers demonstrated additive inhibitory effects for cocktail antibodies [18]. The crystal structure of one of the antibodies, B38, has an epitope that largely overlaps with the ACE2 binding interface, which is similar to the epitope of RBD-chAb-25 in our study. Crowe and co-workers also reported the use of cocktail antibodies to increase the protection from SARS-CoV-2 infection in an animal model. A low-resolution EM structure was demonstrated the simultaneous binding of two nAbs to the RBD of SARS-CoV-2 [34]. While our current study provides very similar findings to those above, including the efficacies derived from *in vitro* and *in vivo* neutralization assays, we provide additional information that was hitherto unavailable. First, we determined the atomic structures of two potent nAbs, namely RBD-chAb-25 and -45 in complex with the SARS-CoV-2 S protein, which revealed an unusual 3:3 binding stoichiometry (Fig 4). In other words, both RBD-chAbs occupy all three RBDs to preclude ACE2 binding to the S protein, although RBD-chAb-25 is similar to REGEN10933 (one of the antibodies in the REGN-COV2 cocktail), with regard to its loss of neutralizing ability against SARS-CoV-2 variants B.1.1.7, B.1.351 and P.1 [57,59]. Second, the structure-guided design of the cocktail therapy showed promising therapeutic effects in mouse and hamster models (Fig 8). Based on these structural insights, we predict that recognition of the non-overlapping epitopes for RBD-chAb-25 and -45 would provide improved protection from different SARS-CoV-2 variants, including the emerging United Kingdom and South African variants. In particular, the epitope of RBD-chAb-45 is less utilized by other reported nAbs, making it an ideal candidate for use in antibody cocktail therapies.

## Materials and methods

### Ethics statement

All animal experiments were performed according to established guidelines for the ethical use and care of animals provided by the Institutional Animal Care and Use Committee (IACUC) at Academia Sinica, Taiwan. All experiments involving animals were approved by the IACUC (protocol 20-05-147). Mice and hamsters were housed individually in cages on a 12-hr light/dark cycle at 20–24°C and given free access to food and water. In order to minimize suffering, animals were euthanized upon loss of over 20% body weight or when the animal exhibited hunching, lack of movement, ruffled fur, and poor grooming. The mice were killed by CO_2_ asphyxiation.

### Construction and purification of RBD and SARS-CoV-2 S recombinant protein

The DNA fragments encoding the RBD, amino acid residues Arg319-Phe541 of SARS-CoV-2 S protein, were amplified by PCR with PfuTurbo DNA polymerase (Stratagene). The PCR products were then cloned into pcDNA3.4-Flag-His vector with IgGκ signal sequence to generate pcDNA3.4-S1-Flag-His and pcDNA3.4-RBD-Flag-His. The RBD-Flag-His protein was produced using Expi293F Expression System (Thermo Fisher Scientific) and purified by Ni Sepharose (GE Healthcare Bio-sciences), followed by anti-Flag M2 agarose beads (Sigma). The DNA fragments were also cloned into a pcDNA3.4-Fc vector with IgGκ signal sequence to generate pcDNA3.4-RBD-Fc, respectively. The RBD-Fc protein was produced using Expi293F Expression System (Thermo Fisher Scientific) and purified by Protein G Sepharose 4 Fast Flow (GE Healthcare) according to the manufacturer’s instructions.

The codon-optimized nucleotide sequence of full-length SARS-CoV-2 S protein was kindly provided by Dr. Che Alex Ma (Genomics Research Center, Academia Sinica). The DNA sequence corresponding the residues 1–1208 of the S protein was subcloned into the mammalian expression vector pcDNA3.4-TOPO (Invitrogen). Additional mutations were introduced for stabilization [60], namely a polybasic furin cleavage site mutation (^682^RRAR^685^ → ^682^GSAG^685^) and a tandem proline substitution (^986^KV^987^ → ^986^PP^987^), hereafter designated as SARS-CoV-2 S_fm2P_. The construct harbors a C-terminal foldon trimerization domain of phage T4 fibritin followed by a c-myc epitope and a hexa-repeat histidine tag for affinity purification.

SARS-CoV-2 S_fm2P_ was transiently transfected into HEK293 Freestyle cells with polyethylenimine (PEI, linear, 25 kDa, Polysciences, U.S.A.) at a ratio of DNA: PEI = 1:2. The transfected cells were incubated at 37°C with 8% CO_2_ for six days. After pelleting the cell culture by centrifugation at 4000 rpm for 30 min, the medium was harvested and filtered through a 0.22-μm cutoff membrane (Satorius, France). The cleared medium was incubated with HisPur Cobalt Resin (Thermo Fisher Scientific, U.S.A.) in 50 mM Tris-HCl (pH 7.6), 300 mM NaCl, 5 mM imidazole and 0.02% NaN_3_ at 4°C overnight. The resin was washed with wash buffer (50 mM Tris-HCl (pH7.6), 300 mM NaCl, 10 mM imidazole), and the target protein was subsequently eluted by 50 mM Tris-HCl (pH 7.6), 150 mM NaCl, 150 mM imidazole. The protein was concentrated and loaded into Superose 6 increase 10/300 GL (GE Healthcare, U.S.A.) in 50 mM Tris-HCl (pH7.6), 150 mM NaCl, 0.02% NaN_3_ for further purification. The concentration of S protein was determined by using the UV absorbance at 280 nm using a UV-Vis spectrometer (Nano-photometer N60, IMPLEN, Germany).

### Screening and binding of antibodies against SARS-CoV-2 by ELISA

The ELISA plates were coated with 0.5 μg/ml RBD-His, S-His, or EpEX-His protein in 0.1 M NaHCO_3_ (pH 8.6) buffer at 4°C overnight, followed by blocking with PBS containing 1% bovine serum albumin (BSA) at RT for 2 h. After blocking, the wells were washed twice with PBS; the plates were then stored at -20°C.

The protein contents of the culture supernatants from hybridoma or antibodies were quantified by the BCA assay and serially diluted with 1% BSA in PBS. Then, 50 μl supernatant or antibody was added into each well, and the plate was incubated for 1 h at room temperature. The plates were washed with PBS containing 0.1% Tween-20 (PBST_0.1_) three times and then incubated for 1 h with Peroxidase AffiniPure Goat Anti-mouse IgG (H+L) (Jackson ImmunoResearch) or Peroxidase AffiniPure Goat Anti-human IgG (H+L) (Jackson ImmunoResearch) (1:5000 dilution), as appropriate. After three washes with PBST_0.1_, signal was produced using 3,3’5,5’-Tetramethylbenzidine (TMB) color development (TMBW-1000-01, SURMODICS). The reaction was stopped with 3 N HCl, and absorbance was measured at 450 nm by ELISA reader (Versa Max Tunable Microplate Reader; Molecular Devices).

### Histological analysis

Viral antigen detection in SARS-CoV-2 animal models was accomplished by immunofluorescence staining. The lung was fixed with 4% paraformaldehyde, paraffin embedded and cut into 3-μm sections. Slides were deparaffinized and rehydrated, then incubated with PBS/0.02% Triton X-100 and blocked with 5% BSA at room temperature for 1 h. The anti-SARS-CoV-2 N protein antibody was added to the sections, followed by washing and incubation with Alexa Fluor 568 goat-anti-human IgG (Invitrogen) at 1:200 dilution. After washing in PBS, slides were stained with DAPI (Invitrogen) at 1:100 dilution. The images were acquired using a ZEN 2011 Black Edition (Carl Zeiss MicroImaging GmbH) and LSM 700 confocal microscopy (Carl Zeiss AG).

### Construction and expression of chimeric antibodies (chAbs)

The V_H_ and V_K_ gene segments of mAbs were introduced via appropriate restriction enzyme sites and amplified by PCR with KAPA HiFi DNA polymerase (Roche). The V_H_ genes were cloned separately in-frame into a modified expression vector with a signal peptide and human IgG1 constant region. The V_L_ genes were also separately cloned into a modified expression vector with a signal peptide and human kappa chain constant region. The V_H_- and V_L_-encoding plasmids were co-transfected into Expi-293 cells, which were cultured for 5 days to produce antibodies. The culture supernatant from the transfected cells was filtered through a 0.45-μm membrane and then subjected to protein G column chromatography (GE healthcare) for purification of human IgG. After the dialysis of eluents with PBS, the antibody concentration was assessed using the Bradford assay (Thermo Fisher Scientific).

### Pseudovirus neutralization assay

The pseudovirus neutralization assays were performed using ACE2-overexpressed 293T cells. Various concentrations of chAbs were mixed with SARS-CoV-2 or SARS-CoV-2 D614G mutant pseudovirus with 1000 TU/well in 96-well plates. The mixture was incubated for 1 h at 37°C and then added to pre-seeded 293T cells at 100 μl/well for 24 h at 37°C. The supernatants were removed after 24 h and refilled with 100 μl/well DMEM for additional 72-h incubation. Next, 100 μl of supernatants were removed, and 100 μl ONE-Glo luciferase reagent (Promega) was added to each well for 3-min incubation. The luciferase activities were measured with a microplate spectrophotometer (Molecular Devices). The inhibition rate was calculated by comparing the OD value to the negative and positive control wells. IC_50_ and IC_80_ were determined by a four-parameter logistic regression using GraphPad Prism (GraphPad Software Inc.).

### Plaque reduction neutralization test (PRNT)

Serially diluted chAbs were incubated with 100 PFU SARS-CoV-2 (strain: TCDC#4) for 1 h at 37°C. The virus-mAb mixtures were added to pre-seeded Vero E6 cells for 1-h adsorption at 37°C; each experiment was performed in triplicate. The viral mixtures were removed and overlaid with DMEM containing 2% FBS and 1% methyl-cellulose. After 4-day incubation, the cells were fixed with 10% formaldehyde overnight and stained with 0.5% crystal violet for 20 min. The plates were washed with tap water, and plaque numbers were counted. Plaque reduction was calculated as: Inhibition percentage = 100 × [1 - (plaque number incubated with mAb/plaque number without mAb)]. The 50% plaque reduction (PRNT_50_) titer was calculated by Prism software. The SARS-CoV-2 used in this study, the clinical isolate TCDC#4 (hCoV-19/Taiwan/4/2020), was obtained from Taiwan Centers of Disease Control (CDC). The PRNT assay was performed at the BSL-3 facility in the Institute of Biomedical Sciences, Academia Sinica.

### Equilibrium dissociation constant (*K*_*D*_) of SARS-CoV-2-RBD binding to chAbs

Binding kinetic measurements were performed using a Biacore 8K (GE Healthcare). All assays were performed with a running buffer of PBS pH 7.4 supplemented with 0.005% (v/v) Surfactant P20 (GE Healthcare) at 25°C. Anti-RBD chimeric antibodies were immobilized onto a protein A sensor chip surface to a level of ~180 response units (RUs). SARS-CoV-2 RBD-His protein was injected in a two-fold dilution series from 40 nM to 0.625 nM, at a flow rate of 50 μl/min using a Multi-cycle kinetics program with an association time of 150 sec and a dissociation time of 300 sec. Running buffer was also injected using the same program for background subtraction. *K*_*D*_ values (affinity constant or dissociation equilibrium constant) were calculated from all the binding curves based on their global fit to a 1:1 binding model by Biacore 8k data analysis software.

### Site-directed mutagenesis of ACE2-binding residues within the RBD

The K417, Y453, Q474, F486, Q498, T500, and N501 residues within the RBD of S protein are responsible for its interaction with ACE2 [28], and each ACE2-binding residue was individually replaced with alanine by site-directed mutagenesis. Mutagenesis was performed using KAPA HiFi Polymerase (Kapa Biosystems) and DpnI digestion, according to the manufacturer’s instructions. RBD mutants were constructed with a single mutation at each ACE2-binding residue or multiple mutations if the residues were neighbors. All mutant constructs were confirmed by sequencing.

### Epitope mapping by ELISA

RBD-chAbs were biotin-labeled using EZ-Link Sulfo-NHS-Lc-Biotin (Thermo; following manufacturer recommendations) and purified using an Amicon Ultra-0.5 Centrifugal Filter Unit (Millipore). Each RBD-chAb (50 ng/well) was pre-coated to ELISA plates. RBD-His or EpEx-His protein (5 ng/well) in bovine serum albumin (BSA) was added to capture Ab-pre-coated ELISA plates, followed by the addition of RBD-chAb (7.8 ng/well) in BSA. Then plates were added biotinylated antibodies (0.78 ng/well) in BSA and incubated at 25°C for 1 h, and 50 μl of 2000-fold diluted Peroxidase Streptavidin (Jackson) was added into each well and incubated for 1 h at 25°C. The BSA without biotinylated antibodies was as a control. The plates were washed with PBST between each step. After a final wash, the plates were developed with TMB, and absorbance was read at 450 nm after the reaction was stopped.

### *In vivo* prophylactic and therapeutic assays for SARS-CoV-2 infection

To assess the *in vivo* potency of neutralizing chAbs against SARS-CoV-2 RBD, mouse and hamster models of SARS-CoV-2 infection were utilized. AAV-hACE2 mice were prepared by intratracheal injection of AAV6 expressing hACE2 and intraperitoneal injection of AAV9 expressing hACE2 (manuscript in submission). The AAV-hACE2-transduced mice or hamsters were first given an intraperitoneal injection of antibody or normal mouse IgG. Intranasal inoculations of 10^5^ tissue-culture infectious dose (TCID) SARS-CoV-2 (strain: TCDC#4) were administered to mice or 10^5^ plaque-forming units (PFU) were administered hamsters 24 h later. Five days or 3 days after the virus challenge to mice or hamsters, lung tissues were harvested to quantify the viral load. Lung tissues were weighed and homogenized using the SpeedMill PLUS (Analytik Jena AG) for two rounds of 2 min each in 0.6 ml of DMEM with 1% penicillin/streptomycin or RLT buffer (RNeasy mini kit, Qiagen). Homogenates were centrifuged at 3,000 rpm for 5 min at 4°C. The supernatant was collected and stored at -80°C for TCID_50_ assay or RNA extraction. After tissue homogenization, serial 10-fold dilutions of each sample were inoculated in a Vero-E6 cell monolayer in quadruplicate and cultured in DMEM with 1% FBS and penicillin/streptomycin. The plates were observed for cytopathic effects for 4 days. TCID_50_ was interpreted as the amount of virus that caused cytopathic effects in 50% of inoculated wells. Virus titers are expressed as TCID_50_/ml tissue.

The *in vivo* assays to assess therapeutic activities of chAbs cocktails were conducted by intraperitoneally injecting mixtures of RBD-chAB-25 and -45. AAV-hACE2 mice or hamsters were intranasally infected with 1 × 10^5^ TCID_50_ virus. Then, antibodies were intraperitoneally injected into mice or hamsters at day 2 after SARS-CoV-2 inoculation. The mice or hamsters were sacrificed to collect tissue and blood samples at day 5 or 3 post-infection, respectively.

### *In vivo* prophylactic assays for low dose of neutralizing mAbs against SARS-CoV-2 infection in hamsters

To assess the *in vivo* potency of low dose neutralizing mAbs against SARS-CoV-2 RBD, hamster models of SARS-CoV-2 infection were utilized. The hamsters were first given an intraperitoneal injection of antibody or normal mouse IgG. Intranasal inoculations of 10^5^ tissue-culture infectious dose (TCID) SARS-CoV-2 (strain: TCDC#4) were administered to mice or 10^5^ plaque-forming units (PFU) were administered hamsters 3 or 5 days later. Three days after the virus challenge to mice or hamsters, lung tissues were harvested to quantify the viral load. Lung tissues were weighed and homogenized using the SpeedMill PLUS (Analytik Jena AG) for two rounds of 2 min each in 0.6 ml of DMEM with 1% penicillin/streptomycin or RLT buffer (RNeasy mini kit, Qiagen). Homogenates were centrifuged at 3,000 rpm for 5 min at 4°C. The supernatant was collected and stored at -80°C for TCID_50_ assay or RNA extraction. After tissue homogenization, serial 10-fold dilutions of each sample were inoculated in a Vero-E6 cell monolayer in quadruplicate and cultured in DMEM with 1% FBS and penicillin/streptomycin. The plates were observed for cytopathic effects for 4 days. TCID_50_ was interpreted as the amount of virus that caused cytopathic effects in 50% of inoculated wells. Virus titers are expressed as TCID_50_/ml tissue.

### Real-time RT-PCR for SARS-CoV-2 RNA quantification

To quantitate SARS-CoV-2 RNA, primers targeting the envelope (E) gene of SARS-CoV-2 genome were used for Taqman real-time RT-PCR method as previously described [61]. Forward primer E-Sarbeco-F1 (5’-ACAGGTACGTTAATAGTTAATAGCGT-3’) and reverse primer E-Sarbeco-R2 (5’-ATATTGCAGCAGTACGCACACA-3’), in addition to the probe E-Sarbeco-P1 (5’-FAM-ACACTAGCCATCCTTACTGCGCTTCG-BBQ-3’) were used. A total of 30 μL RNA solution was collected by using RNeasy Mini Kit (QIAGEN, Germany) according to the manufacturer’s instructions. 5 μL of RNA sample was added in a total 25 μL mixture using Superscript III one-step RT-PCR system with Platinum Taq Polymerase (Thermo Fisher Scientific, USA). The final reaction mix contained 400 nM forward and reverse primers, 200 nM probe, 1.6 mM of deoxy-ribonucleoside triphosphate (dNTP), 4 mM magnesium sulphate, 50 nM ROX reference dye and 1 μL of enzyme mixture from the kit. The cycling conditions were performed with a one-step PCR protocol: 55°C for 10 min for cDNA synthesis, followed by 3 min at 94°C and 45 amplification cycles at 94°C for 15 sec and 58°C for 30 sec. Data was collected and calculated by Applied Biosystems 7500 Real-Time PCR System (Thermo Fisher Scientific, USA). A synthetic 113-bp oligonucleotide fragment was used as a qPCR standard to estimate copy numbers of viral genome. The oligonucleotides were synthesized by Genomics BioSci and Tech Co. Ltd. (Taipei, Taiwan).

### Cryo-EM sample preparation and data collection

To prepare S-mAb complexes, purified recombinant SARS-CoV-2 S_fm2P_ was mixed individually with RBD-chAb-45, chAb-25, and chAb-15 at a molar ratio of 1:1.4 at room temperature for 1 h. The mixture of was loaded into a size-exclusion column (Superose 6 increase 10/300 GL, GE Healthcare, U. S. A.) to separate the S-mAb complex from free mAbs. Fractions corresponding to the S-mAb complex were confirmed by SDS-PAGE and concentrated to 1 mg/ml for cryo-grid preparation. To collect the ternary complex of S protein in complex with RBD-chAb-25 and -45, the SEC fractions corresponding to the ternary complex were collected, as described in the following section, and concentrated to 1 mg/ml for cryo-grid preparation. Three microliters of each sample were applied onto 300-mesh Quantifoil R1.2/1.3 holey carbon grids. The grids were glow-charged at 20 mA for 30 sec. After 30-sec incubation, the grids were blotted for 2.5 sec at 4°C and 100% humidity, and vitrified using a Vitrobot Mark IV. (ThermoFisher Scientific, U. S. A.).

Cryo-EM data acquisition was performed on a 300 keV Titan Krios transmission electron microscope (ThermoFisher Scientific, U. S. A.) equipped with a Gatan K3 direct detector (Gatan, U. S. A.) in a super-resolution mode using the EPU software (ThermoFisher Scientific). Movies were collected with a defocus range of -1.2 to -1.7 μm at a magnification of 81000×, which results in a pixel size of 0.55 Å. A total of 48–50 e^-^/Å^2^ was distributed over 50 frames with an exposure time of 1.8 sec. The datasets were energy-filtered with a slit width of 15–30 eV, and the dose rates were adjusted to 8–10 e^-^/pix/sec.

### Cryo-EM data processing

All 2× binned super-resolution raw movies of each S-chAb complex were subject to Relion-3.0 with dose-weighting and 5×5 patch-based alignment using GPU-based software MOTIONCOR2 [62]. After motion correction, the corrected micrographs were transferred to cryoSPARC v2.14 [63]. Contrast transfer function (CTF) estimation was performed by patch-based CTF. The exposures with "CTF_fit_to_Res" parameters between 2.5 and 4 Å were selected and applied to particle picking. A small subset of micrographs was used for template-free blob picker, followed by iterative rounds of 2D classification for filtering junk particles. The best 2D classes were then used as templates for particle picking on the remaining micrographs. Likewise, the picked particles were cleaned and re-extracted with a box size of 384 pixels.

For each S-mAb complex, the particle images were initially classified by *ab-initio* reconstruction with C1 symmetry (class = 3). The particles and three ab-initio models were used in heterogeneous refinement to generate three distinct classes (class = 3). For both RBD-chAb-25 and -45, the majority of classes corresponded to an all-open state for all three RBDs. Particles within the best class were used for further processing by using non-uniform 3D refinement imposed with C1 symmetry. The overall resolution of the EM map was estimated by the gold-standard Fourier shell correlation (FSC) = 0.143 (Table B in S1 Text). To improve the resolution at the mAb binding interface of S-chAb-25 and S-chAb-45 complexes, a focused refinement procedure was employed. For S-chAb-25, a further local refinement with a focus mask covering the NTD, RBD and chAb-25 was performed in cryoSPARC. For S-chAb-45, the particles of NU-refinement were symmetrically expanded by C3 symmetry, then converted to Relion-3.0 using the pyem script (developed by Daniel Asarnow, https://github.com/asarnow/pyem). A further focus classification with a focus mask corresponding to the RBD and chAb-45 was implemented in Relion. The particles of the best 3D class were selected and transferred to cryoSPARC for another round of local refinement with same focus mask. Focused masks were generated by a combination of UCSF-Chimera [64], cryoSPARC and Relion. The refined cryo-EM maps of the RBD in complex with S-chAb-25 and S-chAb-45 were deposited in the EMDB under the accession codes EMD-31470 and EMD-31471, respectively. The atomic coordinate of the RBD in complex with S-chAb-25 and S-chAb-45 were deposited in the Protein Data Bank (PDB) under the accession codes 7F62 and 7F63, respectively. Local resolution analysis was calculated using ResMap [65]. For the ternary complex of S-chAb-25 and -45, the curated particle images were analyzed by 3D variability analysis within cryoSPARC, as described elsewhere [66], to identify the subclass of structures with the most abundant chAb-25 EM density on the RBD in addition to the well-defined chAb-45 density on each of the three RBDs (S8 Fig).

### Model building and refinement

The atomic model of SARS-CoV-2 S protein in complex with RBD-chAb-25 and -45 were built using Phenix [67] and Coot [68]. An initial coordinate was generated by using the PDB entry 6XLU as a template in Swiss-Model [69]. The atomic models of the Fabs of RBD-chAb-25 and -45 were generated by Swiss-Model using default settings. The atomic coordinates of the S protein and the Fabs of RBD-chAb-25 and -45 were manually fit into the cryoEM map using UCSF-Chimera, UCSF-ChimeraX [70] and Coot. After iterative manual refinement steps, the coordinates were refined by the real-space refinement module within Phenix. N-linked glycans were built by using the extension module “Glyco” within Coot from the asparagine side chains at which additional EM densities were observed. These asparagine residues comply with the rule of the N-glycosylation sequon (N-X-S/T). The final model was assessed by MolProbity [71] in Coot. Statistics of the model refinement are reported in Table B in S1 Text. For the ternary complex of S-chAb-25 and -45, the refined atomic models of S-chAb-25 and S-chAb-45 were individually fit to the cryo-EM map of the ternary complex. Manual fitting of the substructure of Fab in complex with the RBD was carried out within UCSF-ChimeraX, followed by application of the automated volume fitting function within UCSF-ChimeraX. Additional manual adjustments of the Fab of RBD-chAb-45 were carried out by visual inspection to optimize rigid body docking. Structural visualization and representations were accomplished by a combination of UCSF-Chimera, UCSF-ChimeraX, and Pymol (Schrodinger Inc. U.S.A.).

### Size-exclusion chromatography analysis of S+RBD-chAb complex formation

RBD-chAb binding to the SARS-CoV-2 S protein was analyzed by using a gel filtration column (Superose 6 increase 10/300 GL, GE Healthcare, U.S.A.) in 50 mM Tris-HCl (pH 8.0), 150 mM NaCl, 0.02% NaN_3_ at room temperature. RBD-chAb-25 or -45 was mixed with the SARS-CoV-2 S protein (1 mg/ml) at a 1.4:1 molar ratio and incubated at room temperature for 1 h prior to injection into an FPLC system (AKTA UPC10, GE Healthcare, U.S.A.) for size-exclusion chromatography (SEC). Fractions that correspond to the binary complex of the S protein and RBD-chAb-25 or -45 were collected, pooled and concentrated using a 50-ml centrifugal concentrator with a 50-kDa molecular weight cutoff (Millipore, U.S.A.) before addition of the complementary RBD-chAb-45 or -25 followed by 1 h incubation at room temperature to allow formation of a ternary complex. The mixture was analyzed by the same SEC analysis to confirm stable complex formation. The ternary complex formed by incubation of S protein and RBD-chAb-25 followed by the addition of RBD-chAb-45 was collected as elution fractions (10–12 ml total elution volume) and concentrated by the same procedure used for cryo-EM grid preparation.

## Supporting information

S1 Text Supporting Information of Methods and Tables. (DOCX)

S1 Fig Purification of RBD recombinant protein and immunization of SARS-CoV-2 protein to generate antibodies in mice. A. Coomassie Blue-stained SDS-PAGE of RBD-Fc and RBD-Flag-His protein. B. Two mice were immunized with RBD-Fc protein to induce a robust immune response against SARS-CoV-2 RBD. Each assay was performed in triplicate; all data points are shown as the mean ± SD.
(TIFF)

S2 Fig Identification of SARS-CoV-2 RBD-neutralizing Abs. A. The inhibitory activities of antibodies derived from the supernatants of hybridoma cultures were assessed using ACE2-overexpressing 293T cells by flow cytometry. Antibodies were incubated with RBD-His-FITC (2 μg/ml) for 1 h. After incubation, the mixtures were added to ACE2-overexpressing 293T cells for 30 min. The binding profile was analyzed by Thermo Fisher Scientific, Attune NxT flow cytometry. Red asterisks indicate RBD-specific hybridoma clones exhibiting more than 80% inhibition of binding between SARS-CoV-2 RBD and human ACE2 protein. B. PRNT for the neutralization of all SARS-CoV-2 RBD-reactive chAbs. The inhibitory activities of all 12 chimeric antibodies were examined with authentic SARS-CoV-2 in Vero E6 cells. ChAbs were serially diluted in PBS and used to block infection of Vero E6 cells with SARS-CoV-2. Virus without chAb served as control. Plaques formed at each dilution were counted 4 days after virus infection. Red asterisks indicate the six most efficacious neutralizing RBD-chAbs.
(TIFF)

S3 Fig Cross-reactivity of chAbs. A. Characterization of chAbs against S1 proteins from different coronaviruses. Binding of RBD-chAb-1, -15, -25, -28, -45, and -51 to different coronavirus S1 recombinant proteins were detected by ELISA. OD450, optical density at 450 nm. NHIgG, normal human IgG, as negative control. His Ab, as positive control. Each assay of A was performed in triplicate and the data are presented as mean ± SD (n = 3). B. Immunocytochemistry with anti-SARS-CoV-2 RBD-mAbs in RBD-expressing human 293T cells served as a positive control. Cells were fixed with 4% paraformaldehyde, then blocked with 3% BSA for 1 h. RBD-mAb-1, -15, -25, -28–45, or -51 was incubated at 1 μg/mL for 1 h at room temperature. C. Immunohistochemical staining of six major target organs and tissues that are easily damaged by SARS-CoV-2. Human tissue sections were stained with RBD-mAb-1, -15, -25, -28, -45, and -51 at concentrations of 5 μg/ml. Scale bar = 100 μm.
(TIFF)

S4 Fig Confirmation of neutralizing ability for the top six SARS-CoV-2 RBD-reactive chAbs by PRNT. The inhibitory activities of RBD-chAb-1, -15, -25, -28, -45, and -51 were examined with authentic SARS-CoV-2 in Vero E6 cells. chAbs were used at a maximum concentration of 200 ng/ml and seven 2-fold serial dilutions in PBS. Virus without chAb served as a control. Plaques formed at each dilution were counted 4 days after virus infection. Each assay was performed in duplicate or triplicate.
(TIFF)

S5 Fig Biolayer interferometry sensorgrams and kinetics of RBD-chAbs binding to SARS-CoV-2 RBD. RBD-chAb-1, -15, -25, -28, -45, and -51 were examined. Global fitted curves are shown as red lines. The KD values were calculated using a 1:1 binding model.
(TIFF)

S6 Fig Cryo-EM data process workflow for SARS-CoV-2 S in complex with RBD-chAb-25. (TIFF)

S7 Fig Cryo-EM data process workflow for SARS-CoV-2 S in complex with RBD-chAb-45. (TIFF)

S8 Fig **Expanded views of the cryo-EM map to structure model at the RBD-chAb binding interfaces in Fig 4C (A) and 4F (B).** The refined cryo-EM maps of the RBD in complex with S-chAb-25 and S-chAb-45 were deposited in the Electron Microscopy Data Bank (EMDB) under the accession codes EMD-31470 and EMD-31471, respectively. The atomic coordinates of the RBD in complex with S-chAb-25 and S-chAb-45 were deposited in the Protein Data Bank (PDB) under the accession codes 7F62 and 7F63, respectively.
(TIFF)

S9 Fig Verification of non-competitive binding of two RBD-chAbs to SARS-CoV-2 S RBD by SEC analysis. A. Complex formation between RBD and RBD-chAbs applied in different orders. SEC profiles of RBD, neutralizing antibodies and mixtures are overlaid to illustrate apparent molecular weight changes upon complex formation. The chromatography profiles of RBD alone, chAb-25 alone, RBD + chAb-25, and RBD + chAb-25 with subsequent addition of chAb-45 are shown as black, dark red, orange and green, respectively. B. The chromatography profiles of RBD alone, chAb-45 alone, RBD + chAb-45, and RBD + chAb-45 with subsequent addition of chAb-25 are shown as black, purple, fuchsia and blue, respectively. cplx, complex.
(TIFF)

S10 Fig Binding and neutralization assays of chAbs to diverse SARS-CoV-2 mutants. A-B. The binding ability of RBD-chAb to mutant S protein was examined by cellular ELISA. The human 293T cells were separately transfected with the SARS-CoV-2 wild type (WT) or mutant as indicated. OD_450_, optical density at 450 nm. Each assay was performed in triplicate; data are presented as mean ± SD. C. Binding activity of anti-RBD chAbs was determined by ELISA. Mutants of SARS-CoV-2 RBD-His proteins were immobilized on 96-well plates prior to blocking with 1% BSA in PBS and incubated with anti-RBD chAbs at 100 ng/ml. Signal was detected (OD_450_) after labeling with Donkey anti-human IgG-HRP secondary antibody. NHIgG, normal human IgG, as negative control. His Ab, as positive control. Each assay of was performed in triplicate and the data are presented as mean ± SD (n = 3). D-E. Neutralization assay of B.1.1.7 (D) and B.1.351 (E) variants of SARS-CoV2 pseudoviruses with chimeric anti-RBD antibodies. Each assay was performed in triplicate; data points represent the mean. F. Neutralization test for RBD-chAb-25, 45, or both using D614G, B.1.1.7 and B.1.351 variants of SARS-CoV2 pseudoviruses. Each assay was performed in triplicate; data points represent the mean.
(TIFF)

S11 Fig Structural mapping of RBD-chAb-25 and -45 binding interfaces as well as two mutations of concern, K417N and E484K. The binding interfaces of RBD-chAb25 and RBD-chAb-45 are colored orange and magenta, respectively. Two key mutations present in B.1.351 and P.1 lineages are colored with cyan (K417N) and blue (E484K).
(TIFF)
