# Supplementary material for: Structure-guided antibody cocktail for prevention and treatment of COVID-19
Source: PLoS Pathog. 2021 Oct 21;17(10):e1009704. doi: 10.1371/journal.ppat.1009704 (PMC8530329; doi:10.1371/journal.ppat.1009704)
Supplement: S1 Text — (DOCX) [file ppat.1009704.s001.docx]

Supporting Information of Methods and Tables for

**Structure-guided Antibody Cocktail for Prevention and Treatment of COVID-19**

**Appendix supplementary Methods**

Generation of mAbs against SARS-CoV-2.

Construction of mutant S protein using site-directed mutagenesis.

Binding ability of RBD-chAb to human coronavirus S1, SARS-CoV-2 mutant S protein or

SARS-CoV-2 mutant RBD examined by ELISA.

*In vitro* competition assay by flow cytometry.

Histological analysis of RBD-chAb against normal human tissues.

Size-exclusion chromatography analysis of RBD+RBD-chAb complex formation.

**Tables A to C**

Table A. Immunohistochemical staining evaluation of RBD-expression human 293T cell and human organ/tissue.

Table B. Parameters of cryo-EM data collection, processing and model validation.

Table C. Lung pathology score definition.

**Supporting Information of Related References**

**Appendix Supplementary Methods**

**Generation of mAbs against SARS-CoV-2**

Four- to six-week-old female Balb/cJ mice were obtained from National Laboratory Animal Center and used for experiments. The anti-SARS-CoV-2 mAbs were generated by the hybridoma technique based on standard procedures [1], with previously described modifications [2-6]. The mice were sequentially immunized three or four times with RBD-Fc proteins at two-week intervals. Antigens were intraperitoneally injected at 50 μg/mouse using complete Freund’s adjuvant for the first injection and incomplete Freund’s adjuvant for the others. Three weeks after the last immunization, a booster was administered without adjuvant. Four days after the booster dose, splenocytes from the donor mouse and mouse myeloma cells were fused. Then, the cells were gently resuspended in DMEM containing 15% heated-inactivated fetal bovine serum, HAT, and hybridoma growth factor, and plated (200 μl per well) in 96-well culture plates. Supernatants of hybridomas in 96-well plates were first screened by enzyme-linked immunosorbent assay (ELISA). Positive clones were transferred to a new 96-well plate and tested with an immunohistochemistry assay. Those hybridoma clones that were positive for RBD-His and negative for EpEX-His (a negative control) binding were isolated by limited dilution. Ascites were produced in BALB/cJ mice, and the antibodies were purified on Protein G-Sepharose columns (Amersham Biosciences, Piscataway, NJ).

**Construction of mutant S protein using site-directed mutagenesis**

The residues of L18F, R21I, HV69-70del, A222V, N439K, N501Y, D614G and D936Y of S protein are the most frequent mutation events (GISAID; http://gisaid.org). We constructed the mutant S protein by site-directed mutagenesis performed using KAPA HiFi Polymerase (Kapa Biosystems) and DpnI digestion according to the manufacturer's instructions.

**Binding of RBD-chAb to human coronavirus S1, SARS-CoV-2 mutant S protein or SARS-CoV-2 mutant RBD examined by ELISA**

SARS-CoV-2 protein S1-His was purchased from ACRO Biosystems. The other coronavirus S proteins, including SARS-CoV-1 S1-His, MERS-CoV S1-His, 229E-CoV S1-His, HKU-CoV S1-His, OC43-CoV S1-His, NL63-CoV S1-His, were purchased from Sino Biological Inc. SARS-CoV-2 protein RBD (K417N)-His, SARS-CoV-2 protein RBD (K417T)-His, SARS-CoV-2 protein RBD (L452R)-His, SARS-CoV-2 protein RBD (S477N)-His, SARS-CoV-2 protein RBD (E484K)-His and SARS-CoV-2 protein RBD (N501Y)-His were purchased from ACRO Biosystems. For cellular ELISA, HEK-293T cells were transiently transfected with wild-type and mutant S-RA-Flag-His plasmid. The cells were fixed with 4% paraformaldehyde/PBS for 15 min, and then were permeabilized with 0.1% Triton X-100 for 10 min. Cells were blocked using 5% milk, and incubated with RBD-chAb for 1 hr. The binding ability of RBD-chAb to all human coronavirus S1 or SARS-CoV-2 mutant S protein was determined by ELISA analysis. For human coronavirus S1 and RBD recombinant protein-ELISA, plates were coated with 0.5 mg/ml of different subtypes if coronavirus of S1 protein or mutant of RBD proteins in coating buffer. After washing, 100 ng/ml anti-RBD chimeric antibodies or anti-His antibody were added to the wells for 1 hr at room temperature. Then, the horseradish peroxidase-conjugated anti-human antibody (1:8000) and the horseradish peroxidase-conjugated anti-rabbit antibody (1:20000) were added for 1 hr at room temperature, as appropriate. The binding ability of RBD-chAb to all human coronavirus S1 mutants of SARS-CoV-2 RBD proteins were determined by ELISA.

***In vitro* competition assay by flow cytometry**

Recombinant RBD protein was labeled with FITC in a molar ratio of 1:4 with FluoReporte FITC Protein Labeling Kit (Invitrogen Cat: F6434). Mixtures of RBD-FITC and antibodies were incubated at room temperature for 1 h. The RBD/antibodies mixture was incubated with ACE2-overexpressing 293T cells (1 × 10^5^ cells/ well) in a 96-well culture plate at room temperature for 30 min. The cells were then washed with FACS buffer and subjected to Attune NxT Flow Cytometer (Thermo Fisher Scientific). Mock-adsorbed cells were treated similarly and used for background gating. The percentage of positive cells was measured, and the results were analyzed using GraphPad Prism software (GraphPad Software Inc., San Diego CA).

**Histological analysis** **of RBD-chAb against normal human tissues**

To determine appropriate concentrations for the use of mAbs for IHC, immunocytochemistry of RBD protein expressed in human 293T cells was first optimized. The recombinant RBD expression vector was transfected into 293T cells by PolyJet (SignaGen) in 96-well plates. At 48 h post-transfection, the cells were fixed with 4% paraformaldehyde 30 min. The fixed cells or multi-normal human tissue array (FDA999w, US Biomax) sections were incubated with an anti-SARS-Cov-2 RBD mAb for 1 h at room temperature, which was detected using the Super Sensitive IHC Detection System (BioGenex). After 3,3'-Diaminobenzidine (DAB) chromogen staining, the specimens were counterstained with hematoxylin. Images were acquired with a Leica DM6000 microscope.

**Size-exclusion chromatography analysis of RBD+RBD-chAb complex formation**

FPLC was used to verify non-competitive binding of two chAbs to SARS-CoV-2 S RBD. The RBD+RBD-chAb complex formation was carried out with a Superdex 200 Increase 10/300 column (GE Healthcare), which was pre-equilibrated with a solution containing 20 mM Tris-HCl, pH 8.0 and 150 mM NaCl. The RBD-chAb of interest was mixed with a 2.1-fold molar ratio of purified RBD, and the same amount of competing RBD-chAb was then added to RBD+RBD-chAb complex. Complex formation was performed for 2h at 4°C before size-exclusion chromatography (SEC).

Table A. Immunohistochemical staining evaluation of RBD-expression human 293T cell and human organ/tissue.

| **Human organ/tissue** | **RBD-mab-1** | **RBD-mab-15** | **RBD-mab-25** | **RBD-mab-28** | **RBD-mab-45** | **RBD-mab-51** |
| --- | --- | --- | --- | --- | --- | --- |
| **RBD-expression human 293T cells** | **+** | **+** | **+** | **+** | **+** | **+** |
| **Lung** | **-** | **-** | **-** | **-** | **-** | **-** |
| **Liver** | **-** | **-** | **-** | **-** | **-** | **-** |
| **Spleen** | **-** | **-** | **-** | **-** | **-** | **-** |
| **Heart** | **-** | **-** | **-** | **-** | **-** | **-** |
| **Kidney** | **-** | **-** | **-** | **-** | **-** | **-** |
| **Larynx** | **-** | **-** | **-** | **-** | **-** | **-** |
| **Cerebrum** | **-** | **-** | **-** | **-** | **-** | **-** |
| **Cerebellum** | **-** | **-** | **-** | **-** | **-** | **-** |
| **Adrenal gland** | **-** | **-** | **-** | **-** | **-** | **-** |
| **Ovary** | **-** | **-** | **-** | **-** | **-** | **-** |
| **Pancreas** | **-** | **-** | **-** | **-** | **-** | **-** |
| **Parathyroid gland** | **-** | **-** | **-** | **-** | **-** | **-** |
| **Hypophysis** | **-** | **-** | **-** | **-** | **-** | **-** |
| **Testis** | **-** | **-** | **-** | **-** | **-** | **-** |
| **Thyroid gland** | **-** | **-** | **-** | **-** | **-** | **-** |
| **Breast** | **-** | **-** | **-** | **-** | **-** | **-** |
| **Tonsil** | **-** | **-** | **-** | **-** | **-** | **-** |
| **Thymus** | **-** | **-** | **-** | **-** | **-** | **-** |
| **Bone marrow** | **-** | **-** | **-** | **-** | **-** | **-** |
| **Cardiac muscle** | **-** | **-** | **-** | **-** | **-** | **-** |
| **Esophagus** | **-** | **-** | **-** | **-** | **-** | **-** |
| **Stomach** | **-** | **-** | **-** | **-** | **-** | **-** |
| **Small intestine** | **-** | **-** | **-** | **-** | **-** | **-** |
| **Colon** | **-** | **-** | **-** | **-** | **-** | **-** |
| **Salivary gland** | **-** | **-** | **-** | **-** | **-** | **-** |
| **Prostate** | **-** | **-** | **-** | **-** | **-** | **-** |
| **Endometrium** | **-** | **-** | **-** | **-** | **-** | **-** |
| **Uterine cervix** | **-** | **-** | **-** | **-** | **-** | **-** |
| **Skeletal muscle** | **-** | **-** | **-** | **-** | **-** | **-** |
| **Skin** | **-** | **-** | **-** | **-** | **-** | **-** |
| **Peripheral nerve** | **-** | **-** | **-** | **-** | **-** | **-** |
| **Mesothelium** | **-** | **-** | **-** | **-** | **-** | **-** |
| **Retina** | **-** | **-** | **-** | **-** | **-** | **-** |

+, positive staining; -, negative staining

Table B. Parameters of cryo-EM data collection, processing and model validation.

|  | **S．chAb-25** | **S．chAb-45** | |
| --- | --- | --- | --- |
| **Data collection** |  |  | |
| Microscope | Titan Krios  (Gatan K3 Summit camera) | | |
| Voltage (keV) | 300 | | |
| Mode | Counting | | |
| Magnification | 81000x | | |
| Dose rate (e^-^/pix/s) | 8 | | |
| Total dose (e^-^/Å^2^) | 48 | | |
| Frames per movie | 50 | | |
| Defocus range (μm) | 0.8-2.7 | 0.8-2.7 | |
| Pixel size (Å) | 1.1 (2x binned) | | |
| Micrographs collected | 3,444 | 5,195 | |
| Micrographs used | 3,010 | 4,840 | |
| Final used particles | 102,537 | 200,245 | |
| Symmetry | C1 | C1 | |
| Map Resolution (Å) | 3.6 | 3.5 | |
|  |  |  | |
| **Model refinement and Validation** | | | |
| ***Model composition*** |  | |  |
| Non-hydrogen atoms | 30,043 | 30,101 |  |
| Protein residues | 3,725 | 3,723 |  |
| Ligands | 75 | 72 |  |
| MolProbity score | 1.92 | 1.76 |  |
| ***Ramachandran (%)*** |  |  |  |
| Favored | 94.02 | 94.61 |  |
| Allowed | 5.93 | 5.25 |  |
| Outliners | 0.05 | 0.14 |  |
| Rotamer outliners (%) | 0.00 | 0.00 |  |
| Clashscore | 9.91 | 7.06 |  |
| ***r.m.s. deviations*** |  |  |  |
| Bond length (Å) | 0.002 | 0.002 |  |
| Bond angles (º) | 0.568 | 0.517 |  |
|  |  |  |  |
| **Deposition** |  |  |  |
| PDB code | 7EJ4 | 7EJ5 |  |
| EMDB code | EMD-30669 | EMD-30670 |  |

Table C. Lung pathology score definition.

| Score | Lung status |
| --- | --- |
| 0 | Normal, no significant finding |
| 1 | Minor inflammation with a slight thickening of alveolar septa and sparse monocyte infiltration |
| 2 | Apparent inflammation, alveolus septa thickening with more interstitial mononuclear inflammatory infiltration |
| 3 | Diffuse alveolar damage, with alveolus septa thickening, and increased infiltration of inflammatory cells |
| 4 | Diffuse alveolar damage, with extensive exudation and septa thickening, shrinking of alveoli, the restricted fusion of the thick septa, obvious septa hemorrhage, and more cell infiltration in alveolar cavities |
| 5 | Diffuse alveolar damage, with massive cell filtration in alveolar cavities and alveoli shrinking, sheets of septa fusion, and hyaline membranes lining the alveolar walls |

**Supporting Information of Related References**

1. Kohler G, Milstein C. Continuous cultures of fused cells secreting antibody of predefined specificity. Nature. 1975;256(5517):495-7. Epub 1975/08/07. doi: DOI 10.1038/256495a0. PubMed PMID: WOS:A1975AL11200037.

2. Liu IJ, Chiu CY, Chen YC, Wu HC. Molecular mimicry of human endothelial cell antigen by autoantibodies to nonstructural protein 1 of dengue cirus. J Biol Chem. 2011;286(11):9726-36. Epub 2011/01/15. doi: 10.1074/jbc.M110.170993. PubMed PMID: WOS:000288247700096; PubMed Central PMCID: PMC3058979.

3. Li PC, Liao MY, Cheng PC, Liang JJ, Liu IJ, Chiu CY, et al. Development of a humanized antibody with high therapeutic potential against Dengue virus type 2. PLoS Negl Trop Dis. 2012;6(5):e1636. Epub 2012/05/09. doi: ARTN e1636

10.1371/journal.pntd.0001636. PubMed PMID: WOS:000304758900019; PubMed Central PMCID: PMC3341331.

4. Tung K-H, Lin C-W, Kuo C-C, Li L-T, Kuo Y-H, Lin C-W, et al. CHC promotes tumor growth and angiogenesis through regulation of HIF-1α and VEGF signaling. Cancer Lett. 2013;331(1):58-67. Epub 2012/12/12. doi: 10.1016/j.canlet.2012.12.001. PubMed PMID: 23228632.

5. Liao MY, Kuo MYP, Lu TY, Wang YP, Wu HC. Generation of an anti-EpCAM antibody and epigenetic regulation of EpCAM in colorectal cancer. Int J Oncol. 2015;46(4):1788-800. Epub 2015/02/06. doi: 10.3892/ijo.2015.2876. PubMed PMID: WOS:000350616400040.

6. Tang CT, Liao MY, Chiu CY, Shen WF, Chiu CY, Cheng PC, et al. Generation of monoclonal antibodies against Dengue virus type 4 and identification of enhancing epitopes on envelope protein. Plos One. 2015;10(8):e0136328. Epub 2015/08/27. doi: ARTN e0136328

10.1371/journal.pone.0136328. PubMed PMID: WOS:000360069400108; PubMed Central PMCID: PMC4550467.
