# Supplementary figures and images for: Structure-guided antibody cocktail for prevention and treatment of COVID-19
Source: PLoS Pathog. 2021 Oct 21;17(10):e1009704. doi: 10.1371/journal.ppat.1009704 (PMC8530329; doi:10.1371/journal.ppat.1009704)

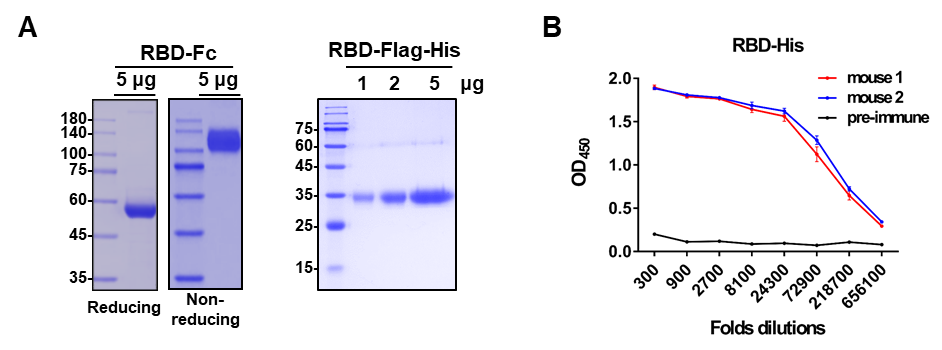

Supplement: S1 Fig — A. Coomassie Blue-stained SDS-PAGE of RBD-Fc and RBD-Flag-His protein. B. Two mice were immunized with RBD-Fc protein to induce a robust immune response against SARS-CoV-2 RBD. Each assay was performed in triplicate; all data points are shown as the mean ± SD. (TIFF) [file ppat.1009704.s002.tiff]

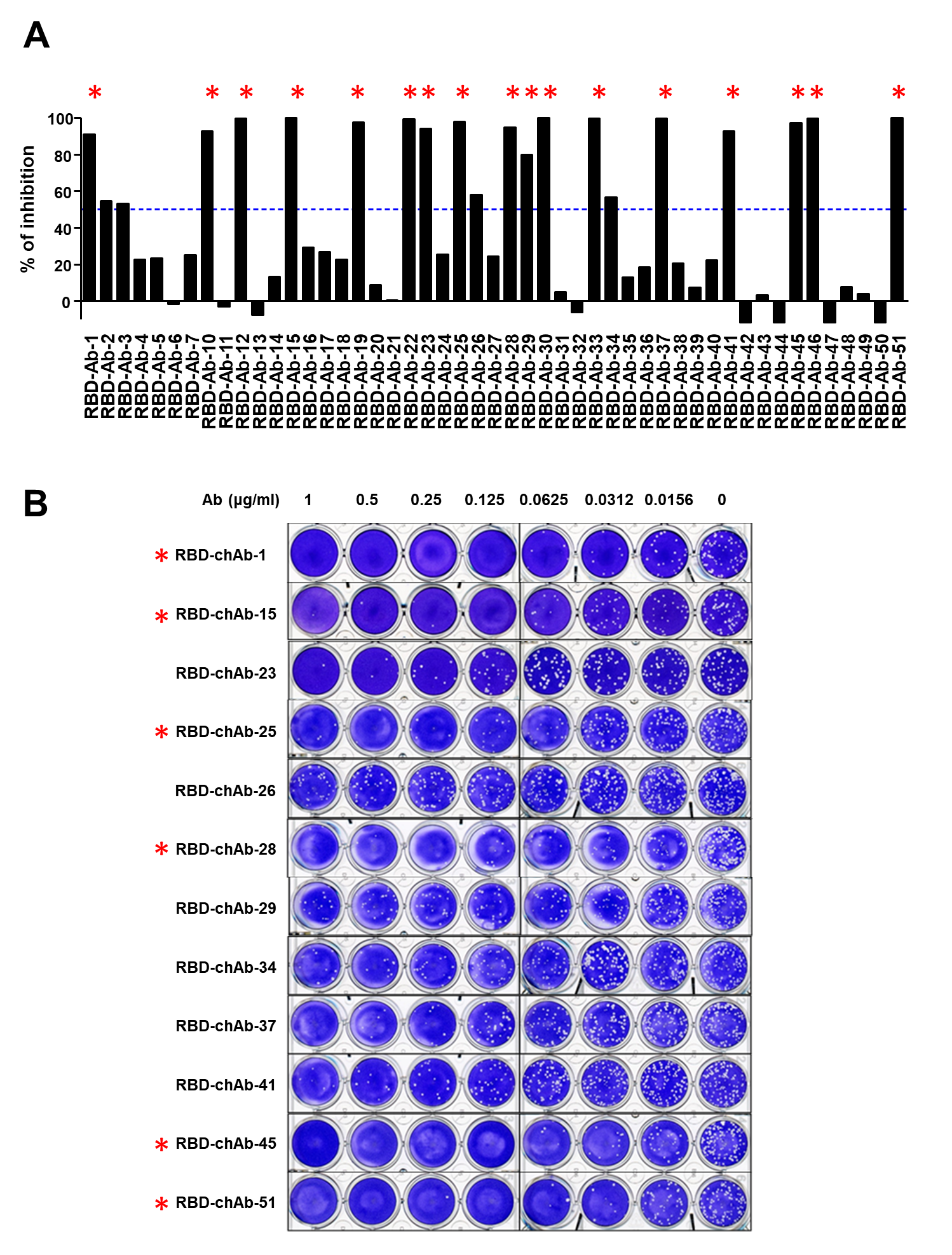

Supplement: S2 Fig — A. The inhibitory activities of antibodies derived from the supernatants of hybridoma cultures were assessed using ACE2-overexpressing 293T cells by flow cytometry. Antibodies were incubated with RBD-His-FITC (2 μg/ml) for 1 h. After incubation, the mixtures were added to ACE2-overexpressing 293T cells for 30 min. The binding profile was analyzed by Thermo Fisher Scientific, Attune NxT flow cytometry. Red asterisks indicate RBD-specific hybridoma clones exhibiting more than 80% inhibition of binding between SARS-CoV-2 RBD and human ACE2 protein. B. PRNT for the neutralization of all SARS-CoV-2 RBD-reactive chAbs. The inhibitory activities of all 12 chimeric antibodies were examined with authentic SARS-CoV-2 in Vero E6 cells. ChAbs were serially diluted in PBS and used to block infection of Vero E6 cells with SARS-CoV-2. Virus without chAb served as control. Plaques formed at each dilution were counted 4 days after virus infection. Red asterisks indicate the six most efficacious neutralizing RBD-chAbs. (TIFF) [file ppat.1009704.s003.tiff]

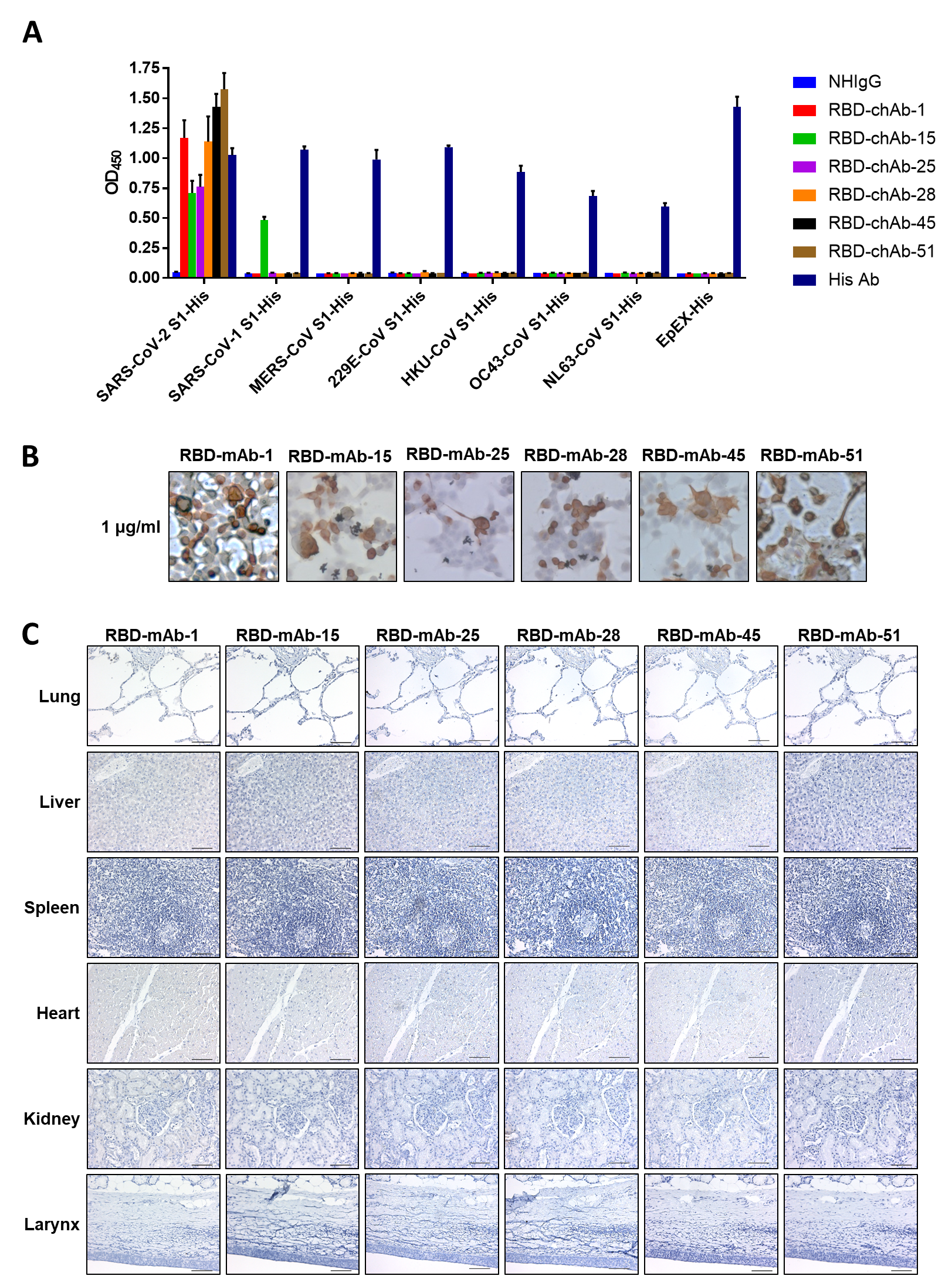

Supplement: S3 Fig — A. Characterization of chAbs against S1 proteins from different coronaviruses. Binding of RBD-chAb-1, -15, -25, -28, -45, and -51 to different coronavirus S1 recombinant proteins were detected by ELISA. OD450, optical density at 450 nm. NHIgG, normal human IgG, as negative control. His Ab, as positive control. Each assay of A was performed in triplicate and the data are presented as mean ± SD (n = 3). B. Immunocytochemistry with anti-SARS-CoV-2 RBD-mAbs in RBD-expressing human 293T cells served as a positive control. Cells were fixed with 4% paraformaldehyde, then blocked with 3% BSA for 1 h. RBD-mAb-1, -15, -25, -28–45, or -51 was incubated at 1 μg/mL for 1 h at room temperature. C. Immunohistochemical staining of six major target organs and tissues that are easily damaged by SARS-CoV-2. Human tissue sections were stained with RBD-mAb-1, -15, -25, -28, -45, and -51 at concentrations of 5 μg/ml. Scale bar = 100 μm. (TIFF) [file ppat.1009704.s004.tiff]

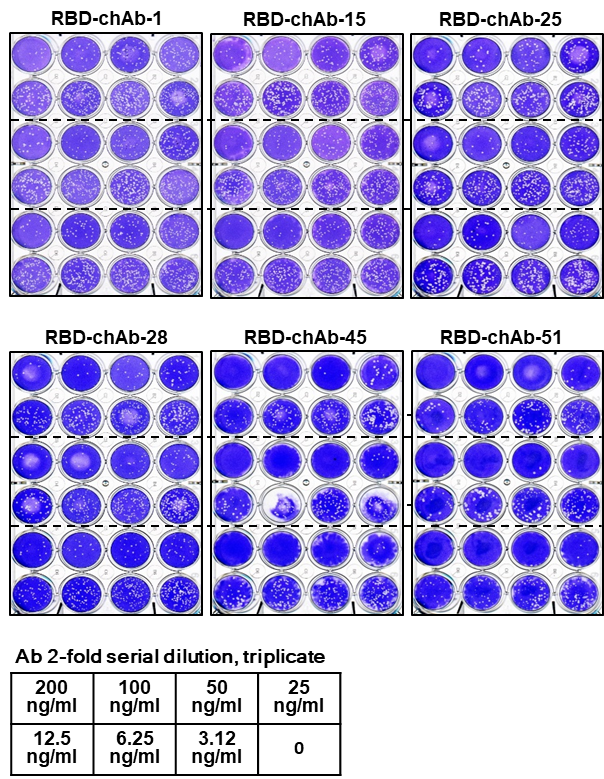

Supplement: S4 Fig — The inhibitory activities of RBD-chAb-1, -15, -25, -28, -45, and -51 were examined with authentic SARS-CoV-2 in Vero E6 cells. chAbs were used at a maximum concentration of 200 ng/ml and seven 2-fold serial dilutions in PBS. Virus without chAb served as a control. Plaques formed at each dilution were counted 4 days after virus infection. Each assay was performed in duplicate or triplicate. (TIFF) [file ppat.1009704.s005.tiff]

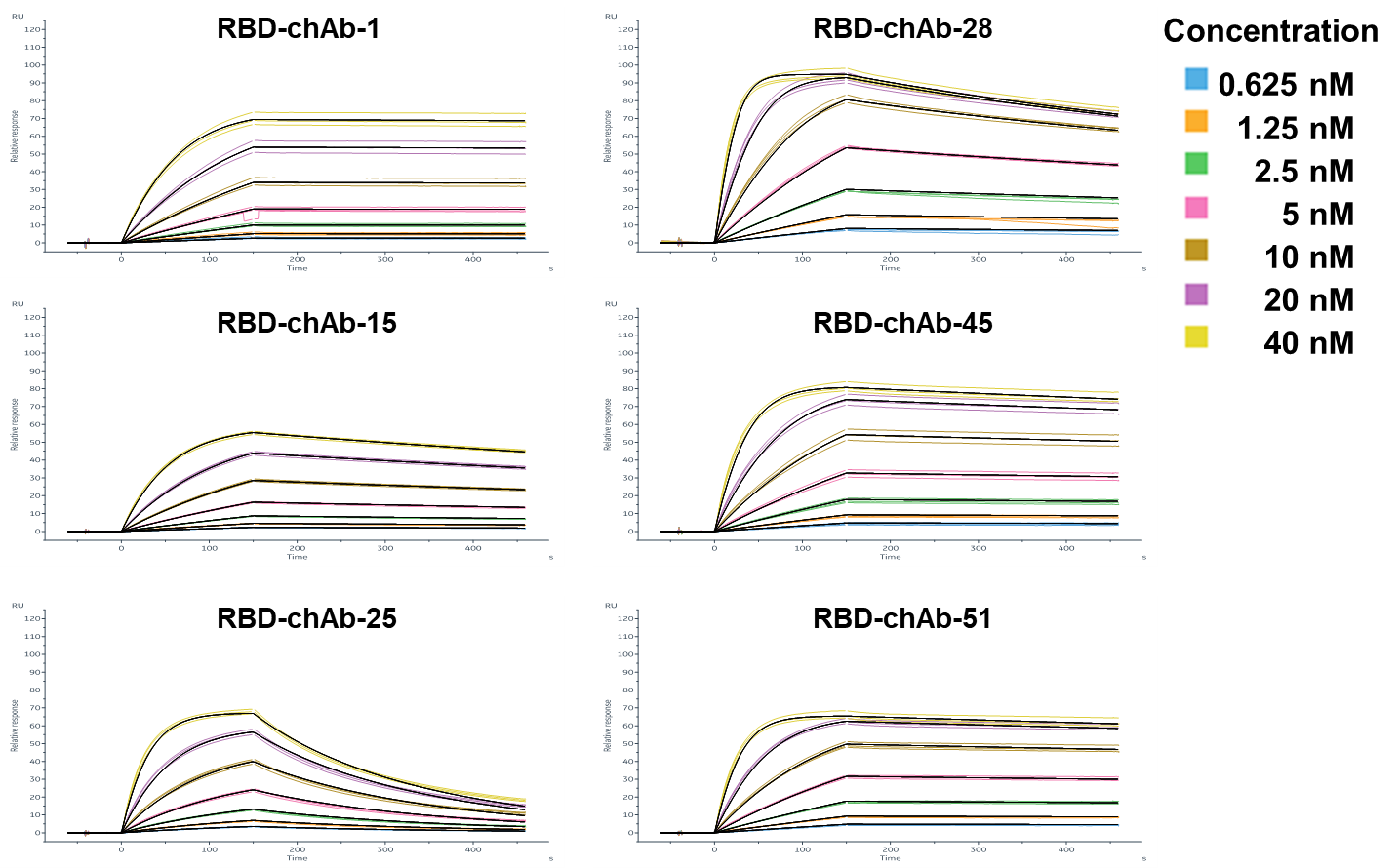

Supplement: S5 Fig — RBD-chAb-1, -15, -25, -28, -45, and -51 were examined. Global fitted curves are shown as red lines. The KD values were calculated using a 1:1 binding model. (TIFF) [file ppat.1009704.s006.tiff]

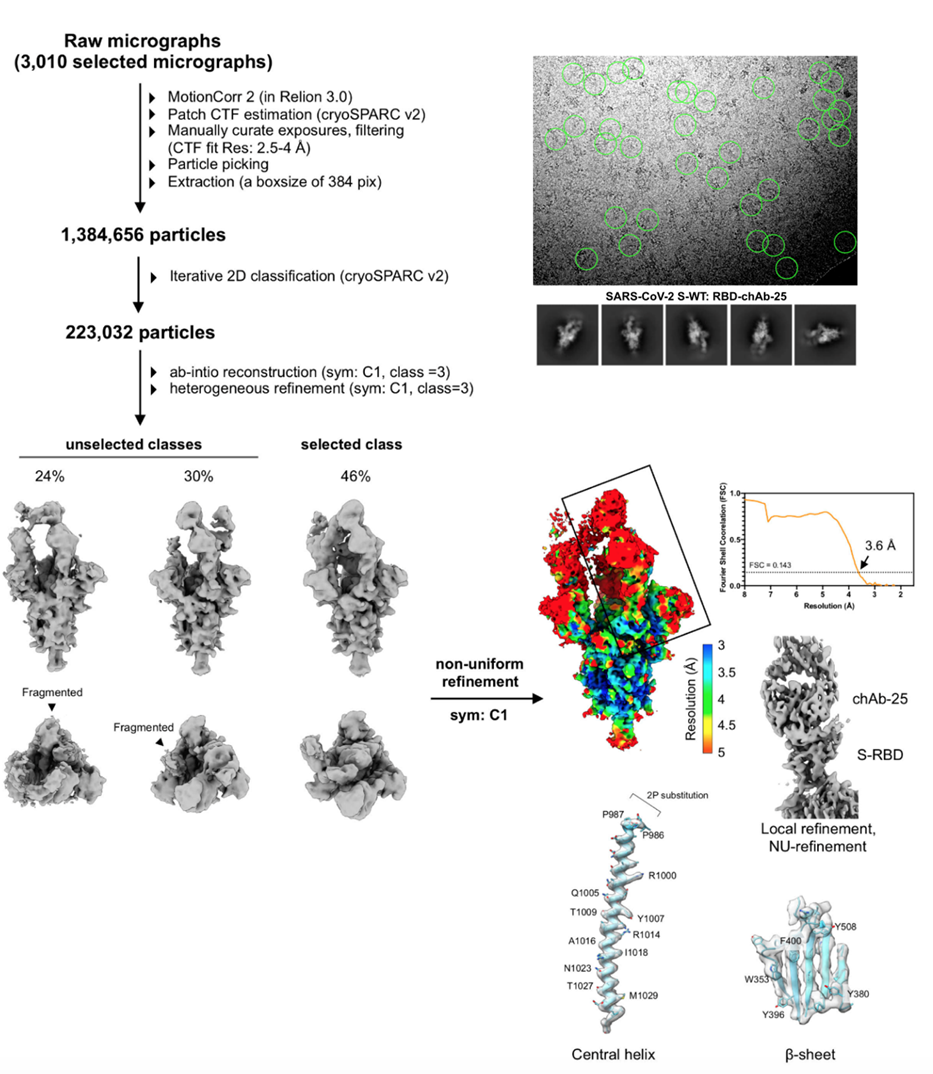

Supplement: S6 Fig — (TIFF) [file ppat.1009704.s007.tiff]

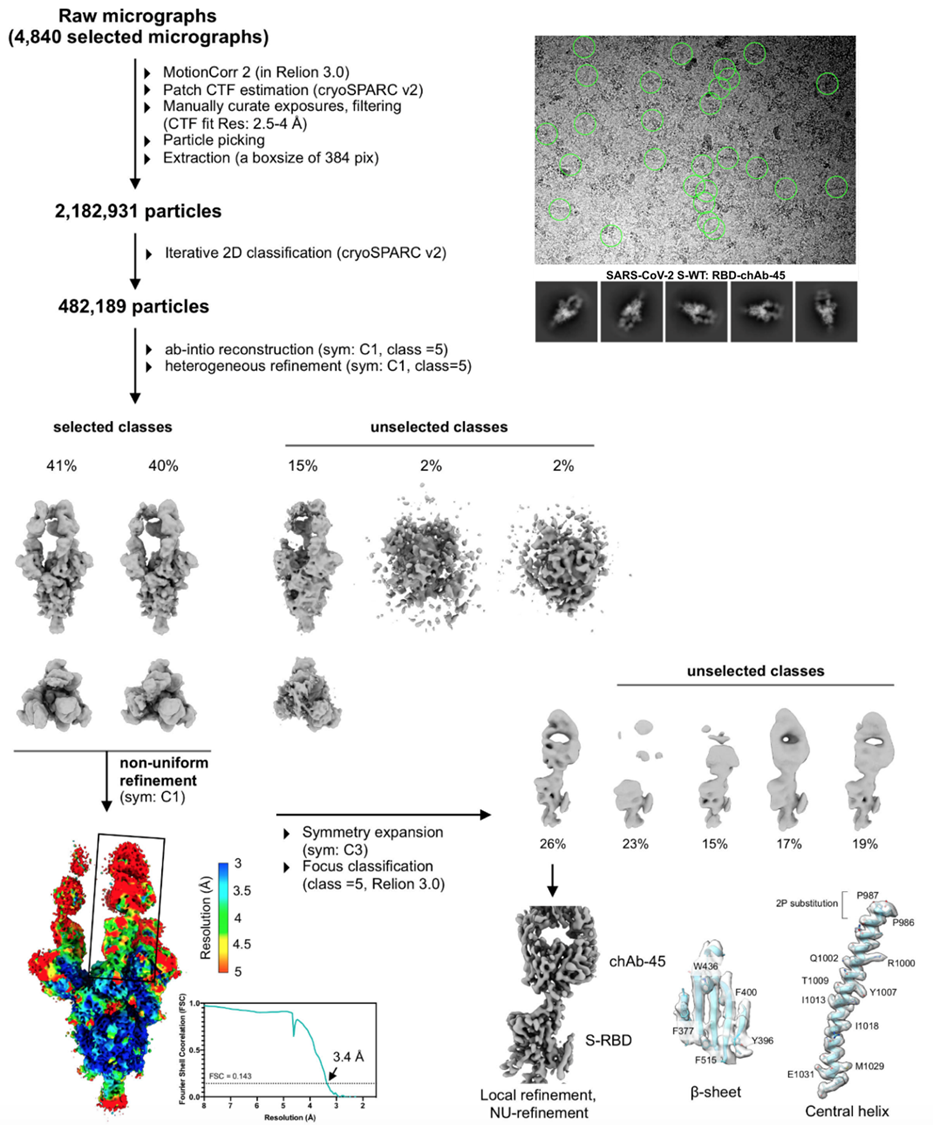

Supplement: S7 Fig — (TIFF) [file ppat.1009704.s008.tiff]

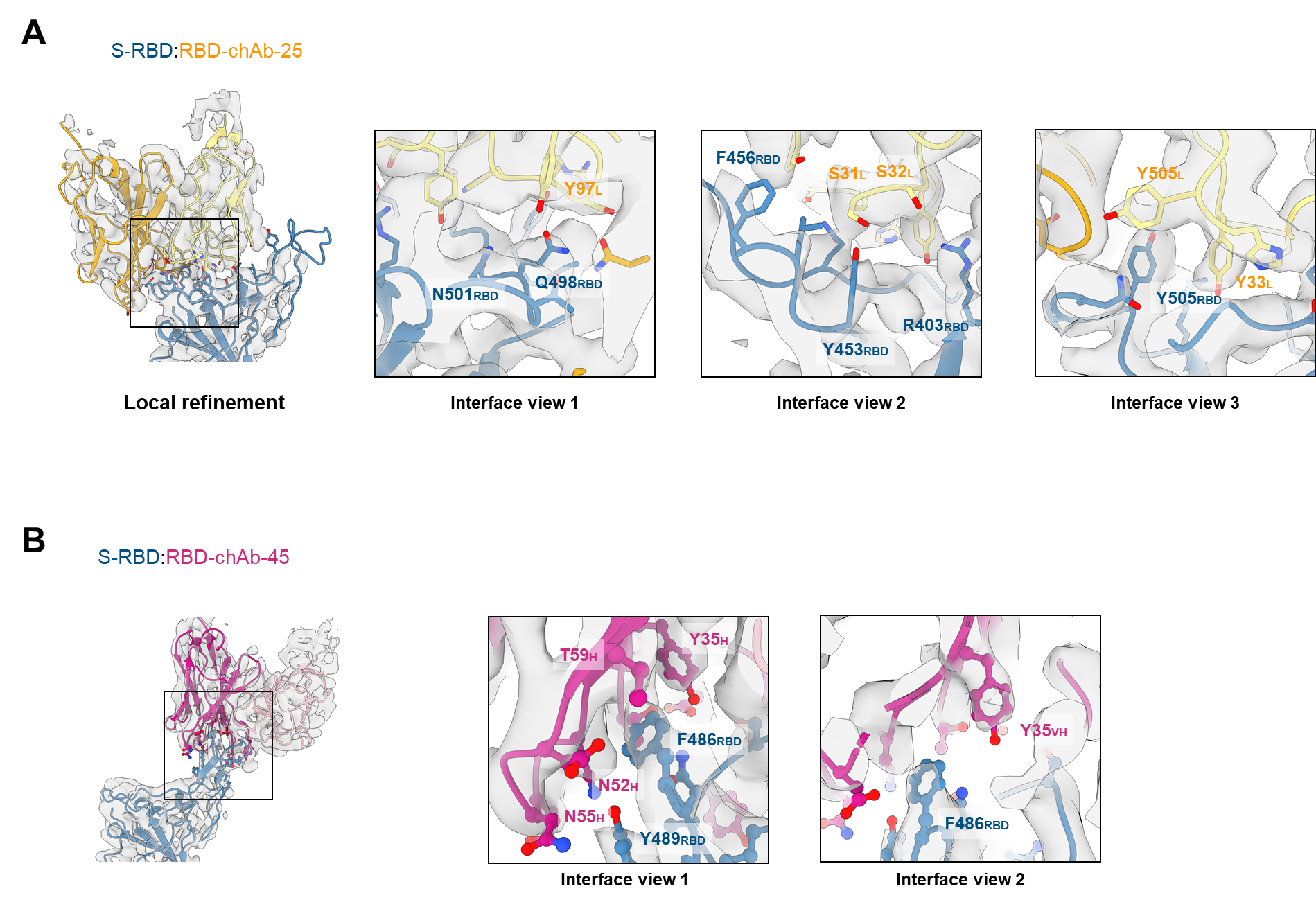

Supplement: S8 Fig — Expanded views of the cryo-EM map to structure model at the RBD-chAb binding interfaces in Fig 4C (A) and 4F (B). The refined cryo-EM maps of the RBD in complex with S-chAb-25 and S-chAb-45 were deposited in the Electron Microscopy Data Bank (EMDB) under the accession codes EMD-31470 and EMD-31471, respectively. The atomic coordinates of the RBD in complex with S-chAb-25 and S-chAb-45 were deposited in the Protein Data Bank (PDB) under the accession codes 7F62 and 7F63, respectively. (TIFF) [file ppat.1009704.s009.tiff]

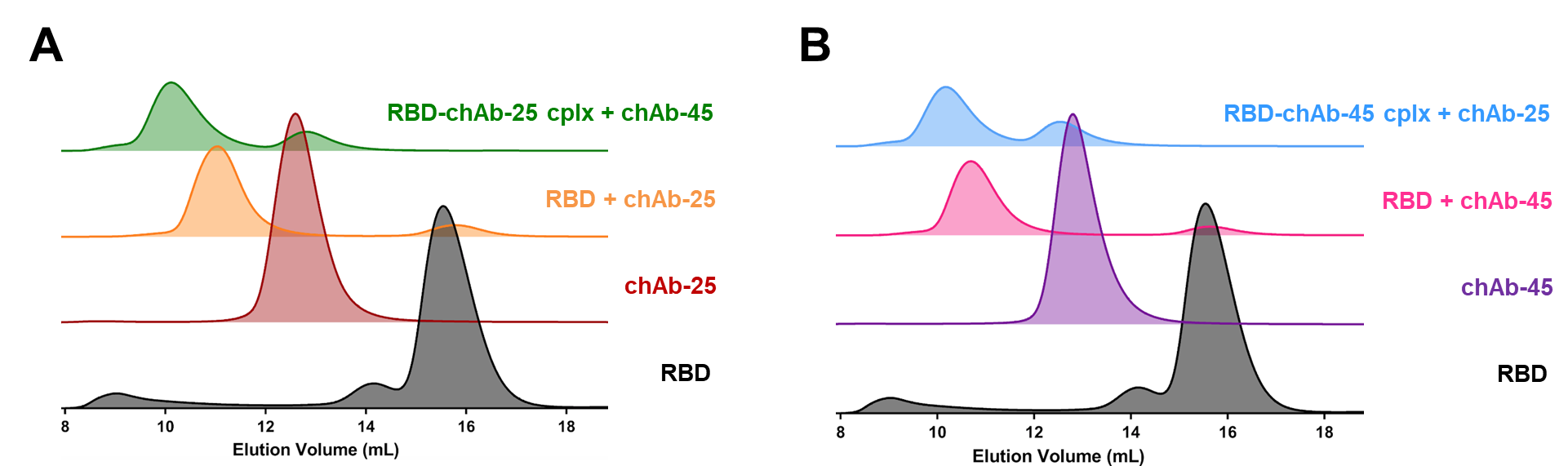

Supplement: S9 Fig — A. Complex formation between RBD and RBD-chAbs applied in different orders. SEC profiles of RBD, neutralizing antibodies and mixtures are overlaid to illustrate apparent molecular weight changes upon complex formation. The chromatography profiles of RBD alone, chAb-25 alone, RBD + chAb-25, and RBD + chAb-25 with subsequent addition of chAb-45 are shown as black, dark red, orange and green, respectively. B. The chromatography profiles of RBD alone, chAb-45 alone, RBD + chAb-45, and RBD + chAb-45 with subsequent addition of chAb-25 are shown as black, purple, fuchsia and blue, respectively. cplx, complex. (TIFF) [file ppat.1009704.s010.tiff]

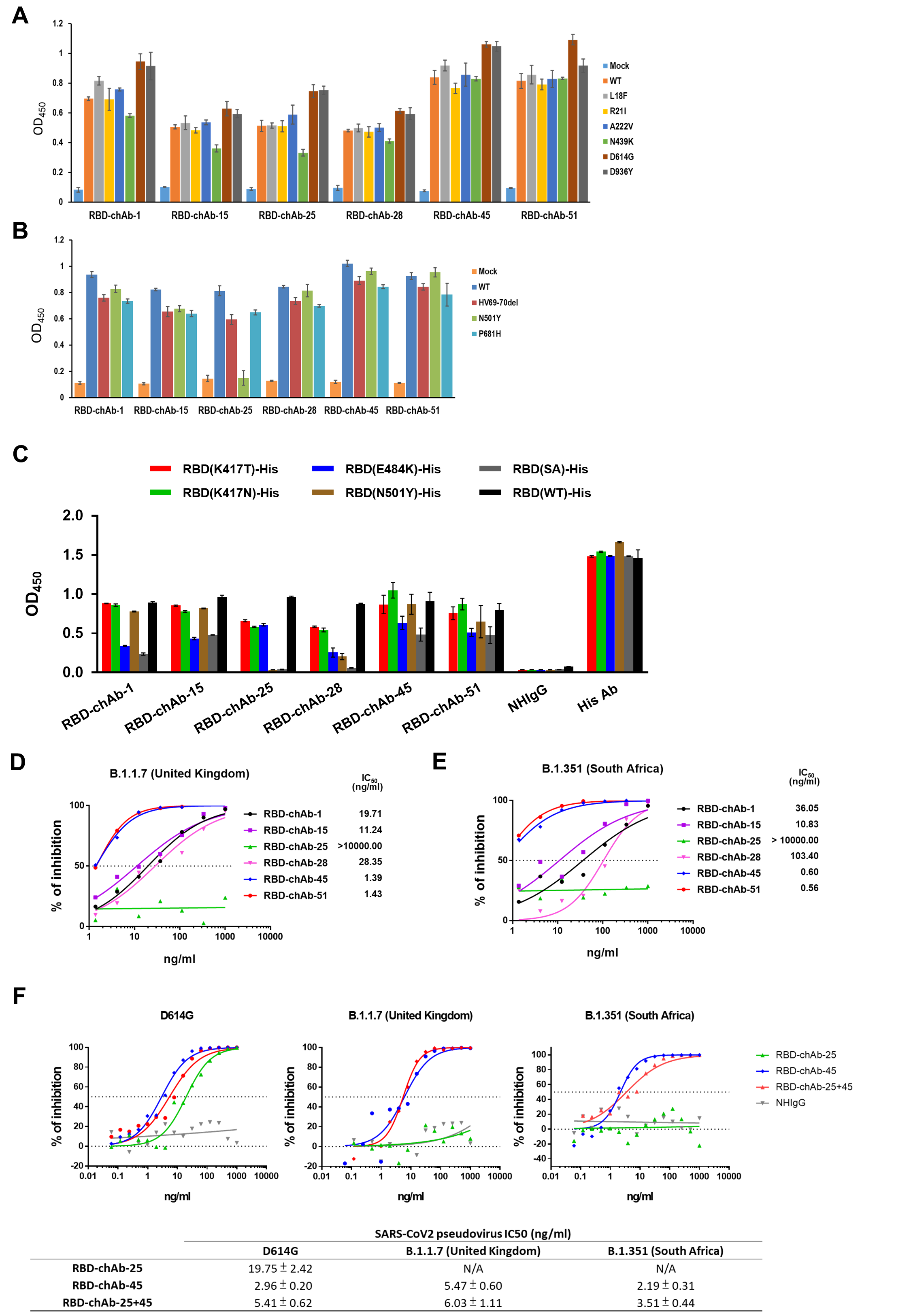

Supplement: S10 Fig — A-B. The binding ability of RBD-chAb to mutant S protein was examined by cellular ELISA. The human 293T cells were separately transfected with the SARS-CoV-2 wild type (WT) or mutant as indicated. OD450, optical density at 450 nm. Each assay was performed in triplicate; data are presented as mean ± SD. C. Binding activity of anti-RBD chAbs was determined by ELISA. Mutants of SARS-CoV-2 RBD-His proteins were immobilized on 96-well plates prior to blocking with 1% BSA in PBS and incubated with anti-RBD chAbs at 100 ng/ml. Signal was detected (OD450) after labeling with Donkey anti-human IgG-HRP secondary antibody. NHIgG, normal human IgG, as negative control. His Ab, as positive control. Each assay of was performed in triplicate and the data are presented as mean ± SD (n = 3). D-E. Neutralization assay of B.1.1.7 (D) and B.1.351 (E) variants of SARS-CoV2 pseudoviruses with chimeric anti-RBD antibodies. Each assay was performed in triplicate; data points represent the mean. F. Neutralization test for RBD-chAb-25, 45, or both using D614G, B.1.1.7 and B.1.351 variants of SARS-CoV2 pseudoviruses. Each assay was performed in triplicate; data points represent the mean. (TIFF) [file ppat.1009704.s011.tiff]

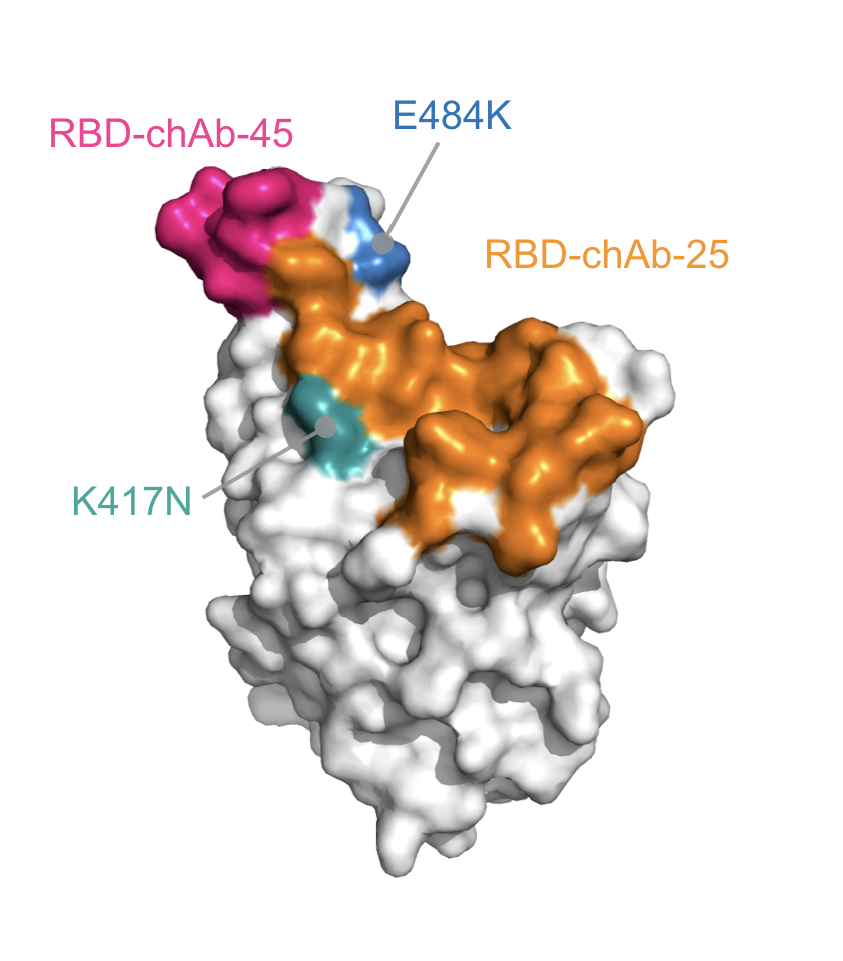

Supplement: S11 Fig — The binding interfaces of RBD-chAb25 and RBD-chAb-45 are colored orange and magenta, respectively. Two key mutations present in B.1.351 and P.1 lineages are colored with cyan (K417N) and blue (E484K). (TIFF) [file ppat.1009704.s012.tiff]
